# Poly (ADP-ribose) polymerases 16 triggers pathological cardiac hypertrophy via activating IRE1α–sXBP1–GATA4 pathway

**DOI:** 10.1007/s00018-023-04805-9

**Published:** 2023-05-23

**Authors:** Haibi Su, Jie Xu, Zhenghua Su, Chenxi Xiao, Jinghuan Wang, Wen Zhong, Chen Meng, Di Yang, Yizhun Zhu

**Affiliations:** 1 School of Pharmacy, Pharmacophenomics Laboratory, Human Phenome Institute, Zhangjiang Fudan International Innovation Center, Fudan University, 825, Zhangheng Road, Pudong New District, Shanghai, 201203 People’s Republic of China; 2 State Key Laboratory of Quality Research in Chinese Medicine and School of Pharmacy, Macau University of Science and Technology, Avenida Wai Long, Taipa, Macau People’s Republic of China

**Keywords:** PARP16, Myocardial hypertrophy, ER stress, GATA4

## Abstract

**Background:**

Pressure overload-induced pathological cardiac hypertrophy is an independent predecessor of heart failure (HF), which remains the leading cause of worldwide mortality. However, current evidence on the molecular determinants of pathological cardiac hypertrophy is still inadequacy. This study aims to elucidate the role and mechanisms of Poly (ADP-ribose) polymerases 16 (PARP16) in the pathogenesis of pathological cardiac hypertrophy.

**Methods:**

Gain and loss of function approaches were used to demonstrate the effects of genetic overexpression or deletion of PARP16 on cardiomyocyte hypertrophic growth in vitro. Ablation of PARP16 by transducing the myocardium with serotype 9 adeno-associated virus (AAV9)-encoding PARP16 shRNA were then subjected to transverse aortic construction (TAC) to investigate the effect of PARP16 on pathological cardiac hypertrophy in vivo. Co-immunoprecipitation (IP) and western blot assay were used to detect the mechanisms of PARP16 in regulating cardiac hypertrophic development.

**Results:**

PARP16 deficiency rescued cardiac dysfunction and ameliorated TAC-induced cardiac hypertrophy and fibrosis in vivo, as well as phenylephrine (PE)-induced cardiomyocyte hypertrophic responses in vitro. Whereas overexpression of PARP16 exacerbated hypertrophic responses including the augmented cardiomyocyte surface area and upregulation of the fetal gene expressions. Mechanistically, PARP16 interacted with IRE1α and ADP-ribosylated IRE1α and then mediated the hypertrophic responses through activating the IRE1α–sXBP1–GATA4 pathway.

**Conclusions:**

Collectively, our results implicated that PARP16 is a contributor to pathological cardiac hypertrophy at least in part via activating the IRE1α–sXBP1–GATA4 pathway, and may be regarded as a new potential target for exploring effective therapeutic interventions of pathological cardiac hypertrophy and heart failure.

**Supplementary Information:**

The online version contains supplementary material available at 10.1007/s00018-023-04805-9.

## Introduction

Cardiac hypertrophy is regarded as an adaptive physiological response of the heart to external stress stimuli, such as pressure overload. However, prolonged cardiac hypertrophy leads to pathological ventricular remodeling and develops into heart failure (HF), which is the final stage of cardiac diseases with high incidence and mortality worldwide [1]. Pathological cardiac hypertrophy is accompanied with enlarged cardiomyocytes area and fetal genes program reactivation, resulting in adverse cardiac remodeling and declined myocardium function [2]. Currently, the combination of angiotensin converting enzyme inhibitors (ACEIs), β-blockers, and aldosterone receptor antagonists are used as the main treatment regimen, however, the overall curative effects are poor. Clearly, new intervention targets are urgently needed to attenuate the progress of pathological cardiac hypertrophy.

ER stress, caused by the accumulation of misfolded or unfolded proteins, is a common feature accompanied with the cardiovascular diseases progression [3]. The unfolded protein response (UPR), which is elicited by ER stress, activates three transmembrane sensors: Protein Kinase R-like ER Kinase (PERK), Inositol Requiring Enzyme 1α (IRE1α) and the Activating Transcription Factor 6 (ATF6) [4]. Among them, IRE1α is the most highly conserved and could splice 26 nucleotides from the un-spliced XBP1 (uXBP1) mRNA, resulting in frameshift and forming the spliced XBP1 (sXBP1), which functions via a variety of transcriptional targets and participates in numerous cellular stress responses [5]. Previous study has elucidated that phospho-IRE1α is increased in TAC-induced hypertrophic heart [6]. In addition, XBP1 is also identified as a novel target for miRNAs such as mir-214, which is closely involved in the progression from adaptive hypertrophy to heart failure [7]. However, the direct association between IRE1α–sXBP1 pathway and pathological cardiac hypertrophy was still unclear.

PARP16, the only member of the Poly (ADP-ribose) polymerase (PARP) family, is correlated with functional activation of ER stress sensors PERK and IRE1α during the UPR [8]. A growing number of studies have identified PARP16 as a potential therapeutic target, such as a unique anticancer target for small cell lung cancer and other tumors [9], but the development of inhibitors against PARP16 is lagging behind. Surprisingly, epigallocatechin-3-gallate (EGCG) was newly characterized as a potential inhibitor of PARP16 and dramatically inhibited its activity in vitro [10]. Furthermore, PARP16 is recently identified as a novel target gene for histone H3 lysine 4 (H3K4) methyltransferase SMYD3. In particular, SMYD3 binds to the promoter of *Parp16* gene and increases H3K4me3 level to activate its host genes' transcription, which finally causes UPR activation and results in neointimal hyperplasia or vascular aging related diseases [11, 12]. However, the effects of PARP16 on the pathological cardiac hypertrophy remain unclear.

In the present study, we determined the role of PARP16 in pathological cardiac hypertrophy. Ablation of PARP16 rescued cardiac dysfunction and ameliorated TAC-induced cardiac hypertrophy and fibrosis in mice, as well as PE-induced cardiomyocyte hypertrophic responses in vitro. Whereas overexpression of PARP16 exacerbated the hypertrophic responses including the augmented cardiomyocyte surface area and upregulation of the hypertrophic markers’ levels. Mechanistically, we found the JNK/ERK MAPK pathway largely contributed to PE-induced PARP16 upregulation, then PARP16 interacted with IRE1α and ADP-ribosylated IRE1α and mediated the hypertrophic responses through activating the IRE1α–sXBP1–GATA4 pathway. Collectively, our results implicated that PARP16 is a contributor of pathological cardiac hypertrophy at least in part via activating the IRE1α–sXBP1–GATA4 pathway. Targeting PARP16 may be an innovative therapeutic strategy to cure pathological cardiac hypertrophy.

## Materials and methods

### Reagents

Reagent sources were as follows: phenylephrine (PE, S224411) and STF-083010 (S81454) were purchased from Yuanye Bio-Technology (Shanghai, China). Tauroursodeoxycholic acid (TUDCA, MB5423) were purchased from Meilun Biotechnology (Dalian, China). Antibodies were obtained from the following commercial sources: ANP (Abcam, ab225844), BNP (Servicebio, GB11667), PARP16 (AVIVA, ARP33751-P050), Hsp90 (Proteintech, 13171-1-AP), β-Tubulin (Proteintech, 66240-1-Ig), MMP2 (Servicebio, GB11130), MMP9 (Servicebio, GB12132-1), Collagen 1 (Proteintech, 14695-1-AP), VCAM-1 (GeneTex, GTX12133), GAPDH (Proteintech, 60004-1-Ig), GATA4 (Santa Cruz Biotechnology, sc-25310), GATA4 (ABclonal Technology, A13756), α-SMA (Servicebio, GB13044), p-IRE1α (ABclonal Technology, AP0878), sXBP-1(Abcam,ab37152), IRE1α (ABclonal Technology, A17940), p-PERK (Cell Signaling Technology, 3179), p-eIF2α (Cell Signaling Technology, 3597), Lamin A/C (Santa Cruz Biotechnology, sc-376248), p-JNK (Abways, CY5541), p-ERK1/2 (Cell Signaling Technology, 4370), p-p38 (Cell Signaling Technology, 4511), Flag (Proteintech, 66008-2-Ig) were used in Western blot assay; ANP (Santa Cruz Biotechnology, sc-18811), BNP (Santa Cruz Biotechnology, sc-18817), α-actinin (Santa Cruz Biotechnology, sc-166524), α-actinin (R&D Systems, AF8279), FN (ABclonal Technology, A7488), PARP16 (AVIVA, ARP33751-P050), GATA4 (Santa Cruz Biotechnology, sc-25310), GATA4 (ABclonal Technology, A13756) were used in immunofluorescence staining.

### Transverse aortic constriction (TAC)-induced pathological cardiac hypertrophy model

TAC was performed to establish the pathological cardiac hypertrophy model. C57BL/6J mice (24–26 g, male) were randomly selected and anesthetized with isoflurane. Then mice were intubated and connected to a volume-cycle rodent ventilator on supplemental oxygen and isoflurane at a rate of 1 L/min and respiratory rate of 140 bpm/min. Next, thoracotomy was performed between the first and second rib. The aortic arch was narrowed against a 27-gauge needle with a 7-0 silk suture ligature. Sham-operated mice were carried out the similar procedure without ligation.

### Intramyocardial delivery of adeno-associated virus (AAV) for infecting mice

Before 2 weeks of TAC operation, intramyocardial delivery of adeno-associated virus (AAV) was performed to infect mice in order to knockdown PARP16. Serotype 9 AAV vectors encoding mouse PARP16 shRNA (AAV9-shPARP16) were constructed by Hanbio Biotechnology (Shanghai, China). Briefly, C57BL/6J mice (6–8 weeks, male) were randomly chosen to receive endotracheal intubation and then connect to the respiratory machine. Open thoraces and expose the heart. Then 50 μL AAV9-shPARP16 (10^11^ particles) or AAV9-GFP were injected into 4–5 sites along the left ventricular walls. Then suture the thoraces. Two weeks later, the GFP fluorescence distribution was observed under a fluorescence microscope (Carl-Zeiss-Promenade 10) to determine the efficiency of adenovirus infection and western blot was performed to examine the knockout efficiency of PARP16.

### Ang II infusion-induced cardiac hypertrophy mouse model

C57BL/6 mice were anesthetized and subcutaneously implanted with the Alzet mini-osmotic pumps (Model 2004, ALZA Scientific Products, Mountain View, CA, USA) containing either saline or Ang II (Meilun Biotechnology, Dalian, China) dissolved in saline. Saline or Ang II (1.5 mg/kg/day) were continuously infused for 4 weeks and mice received injection of either lentivirus PARP16 shRNA (shPARP16) or scramble shRNA(shCTL) every 5 days after the Ang II infusion. The heart sections were harvested for further investigation.

### Echocardiography

The cardiac function of mice at 4 weeks post-TAC was determined by transthoracic echocardiography (VisualSonics, Vevo 2100, Canada). Briefly, the mice were lightly anaesthetized with isoflurane mixed in 1 L/min O_2_ via a facemask. Left ventricular (LV) ejection fraction (EF%), fractional shortening (FS%), the interventricular septal thickness at diastole and systole (IVSd and IVSs, respectively) in the M-mode were measured using the parasternal short-axis view at the level of the papillary muscles. LV mass was estimated by the area length method. EF%, FS% and LV mass were calculated automatically using the VevoLAB program.

### Cell culture and treatment

The primary neonatal rat cardiomyocytes (NRCMs) were isolated from Sprague–Dawley (SD) rats (1–2-day-old). Briefly, hearts were immediately harvested and cut into about 1 mm^2^, then repeatedly digested with 0.25% trypsin (Beyotime Biotechnology, China) at 37 °C for 5 min/cycle. After 8–10 cycles of digestion, the cell suspension was filtered and centrifuged. The collected cells were cultured at 37 °C with 5% CO_2_. After 1.5 h, NRCMs in the supernatant were seeded onto 6-well dishes and cultured in serum-containing medium (DMEM + 10% fetal bovine serum (FBS) + 1% penicillin–streptomycin) and 1% 5-bromodeoxyuridine (BrdU) for 48 h.

The rat myocardial cell line H9C2 (2-1) cells (CL-0089) were purchased from Procell Life Science and Technology Co., Ltd. (Wuhan, China) and cultured in DMEM medium (containing 1.5 g/L NaHCO_3_) supplemented with 10% FBS and 1% penicillin–streptomycin.

NRCMs or H9C2 cells were then administrated with phenylephrine (PE, 100 μM) for 48 h to establish the cardiomyocytes hypertrophy model.

### Small interfering RNA (siRNA) transfection

The rat PARP16-specific short interfering RNA (siPARP16) (sense 5′-CCUACCUCACAAGUGACUUTT-3′, antisense 5′-AAGUCACUUGUGAGGUAGGTT-3′), IRE1α-specific short interfering RNA (siIRE1α) (sense 5′-GGAGCUUUGAGGAGGUUAUTT-3′, antisense 5′-AUAACCYCCYCAAAGCUCCTT-3′) and control siRNA (5′-UUCUCCGAACGUGUCACGUTT-3′) were obtained from GenePharma (Shanghai, China). Cells were transfected with RNA-lipid complexes which contained siRNAs, Lipofectamine RNAiMAX and Opti-MEM according to manufacturer’s instruction.

### Plasmid construction, lentivirus generation and infection

Full length of the rat PARP16 cDNA was cloned into the pCDH-EF1-MSC-T2A-Puro (System Biosciences, CA) lentiviral vector, constructing the PARP16 overexpressing plasmid. The lentiviral vector was named the negative control. Sanger sequencing was used to verify these plasmids.

For the generation of lentivirus, HEK293T cells were co-transfected with three plasmid systems including the recombinant plasmid, and packaging vector psPAX2 and PMD2.G using the transfection reagent Lipofectamine2000 (Invitrogen, USA) for 48 h. The virus stocks were collected and titrated by transduction of H9C2 cells or NRCMs to 1 × 10^7^ TU/mL, which combined with 8 μg/mL polybrene (Sigma) for 24 h. This virus medium was then removed and cells were continued to culture for an additional 48 h in the complete medium.

### Immunofluorescence staining

For the cellular immunofluorescence staining, NRCMs or H9C2 cells were seeded in the glass-bottomed 24-well culture plates. After treatment with different stimulants, the cells were fixed with 4% paraformaldehyde, permeabilized with 0.25% Triton X-100 in PBS for 15 min, blocked with 5% BSA for 30 min and incubated with the appropriate primary antibody at 4 °C overnight. The next day, the cells were stained with the species-specific fluorescent secondary antibody and the nuclei were counter-labeled with 4, 6-Diamidino-2-phenylindole (DAPI, Beyotime Biotechnology, C1005). Images of cardiomyocytes were acquired using a fluorescence microscope.

For measurement of the cardiomyocyte surface area, the Image J software (version 6.0) was used and calculate an average cell surface area in one visual field, then calculating the fold change of each group normalized to the control group.

For the immunofluorescence staining analysis of the heart tissues from the TAC or sham surgery, the hearts were harvested and fixed in 4% paraformaldehyde and then embedded in paraffin. After rehydration and antigen retrieval process, the heart sections were permeabilized with 0.25% Triton X-100 in PBS for 15 min and then blocked with 5% BSA in PBS for 30 min followed by the appropriate primary antibodies. The following steps are consistent with the cellular immunofluorescence staining.

### The morphological parameters detections

The morphological parameters of mice hearts were determined at 4 weeks post-TAC. In brief, the hearts of mice were harvested and fixed in paraformaldehyde (4%) for paraffin-embedding. Then the paraffin sections were exposed to hematoxylin and eosin (H&E) staining for morphological analyses. The cross-sectional areas of cardiomyocytes were calculated via FITC-conjugated wheat germ agglutinin (WGA) staining. The cardiac myocyte cross-sectional areas were measured using Image J software within captured images.

On the other hand, the Sirius Red staining and Masson trichrome staining were performed for collagen deposition assessments. The myocardial fibrosis was measured as a positively stained area with Sirius Red staining and Masson trichrome staining normalized to the total area using Image J software.

### Immunohistochemistry (IHC)

The paraffin-embedded heart slices were blocked for peroxidases using 3% H_2_O_2_-methanol solution for 20 min and incubated with PARP16 antibody at 4 °C overnight. After washing with PBS, heart slices were incubated with anti-rabbit IgG conjugated with peroxidase as second antibody at room temperature (RT) for 1 h, and DAPI was used for counterstaining the nuclear. Representative images were captured under the ZEISS light microscope.

### Western blotting

Left ventricular tissue and cardiomyocytes samples were lysed with RIPA (Pierce, Rockford, IL, USA) lysis buffer containing 1% protease and phosphatase inhibitor cocktail (Sigma, St Louis, USA) or LDS Sample Buffer (Thermo, Invitrogen, CA, USA) and 5% β-mercapto ethanol. The protein concentrations were quantified by BCA protein assay kit (Thermo, Invitrogen, CA, USA). The lysed whole protein samples were separated by SDS-PAGE gels and transferred to nitrocellulose (NC) membranes. The protein bands were incubated with suitable primary antibodies overnight at 4 °C and its corresponding HRP-conjugated secondary antibodies for 1.5 h at RT. Chemiluminescence was generated and detected by ChemiDoc^+^ (Bio-Rad Laboratories, Inc). Protein levels were quantified using Image J software.

### Real-time and quantitative PCR (RT-qPCR) analysis

RT-qPCR analysis was performed as described [13]. Briefly, total RNA from Left ventricular tissue samples or cardiomyocytes were extracted with TRIzol Reagent (TaKaRa Biotechnology, Dalian, China) following the manufacturer’s instructions. Then cDNA was synthesized using a PrimeScript 1st Strand cDNA Synthesis Kit (TaKaRa). The expressions of target genes were quantified by qRT-PCR performed on an iCycler iQ system (Bio-Rad, Hercules, CA, USA) and normalized to GAPDH mRNA. The primer sequences were listed in Table S1.

### Co-immunoprecipitation

The cardiomyocytes and 293T cells were lysed with IP-RIPA lysis buffer. 1 mg of total protein was mixed with 3–5 μg indicated antibodies overnight at 4 °C, then incubated with Protein A/G PLUS-Agarose (sc-2003; Santa Cruz Biotechnology, Santa Cruz, CA) at RT for an additional 2 h. The protein-antibody-bead precipitates were washed for 4 times and then separated by SDS-PAGE for western blotting.

### Extraction of cytosolic and nuclear fraction

In brief, NRCMs or H9C2 cells were treated with PE for 48 h before collection for cytosolic and nuclear fraction extraction assay. The NE-PER Nuclear and Cytoplasmic Extraction Reagents (Thermo Scientific, catalog no. 78835) were used according to the manufacturer’s manual.

### Statistical analysis

The data were analyzed using GraphPad Prism 7.0 (GraphPad Software Inc., San Diego, CA, US) and presented as mean ± SD. Differences of means were analyzed by using one-way ANOVA with Tukey Post-hoc tests for multiple groups, and when comparing between two groups using unpaired Students *t* test. Probability values, *p* < 0.05 was set as statistically significant.

## Results

### PARP16 expresses at a high level in heart tissues and cardiomyocytes

**Fig. 1:**
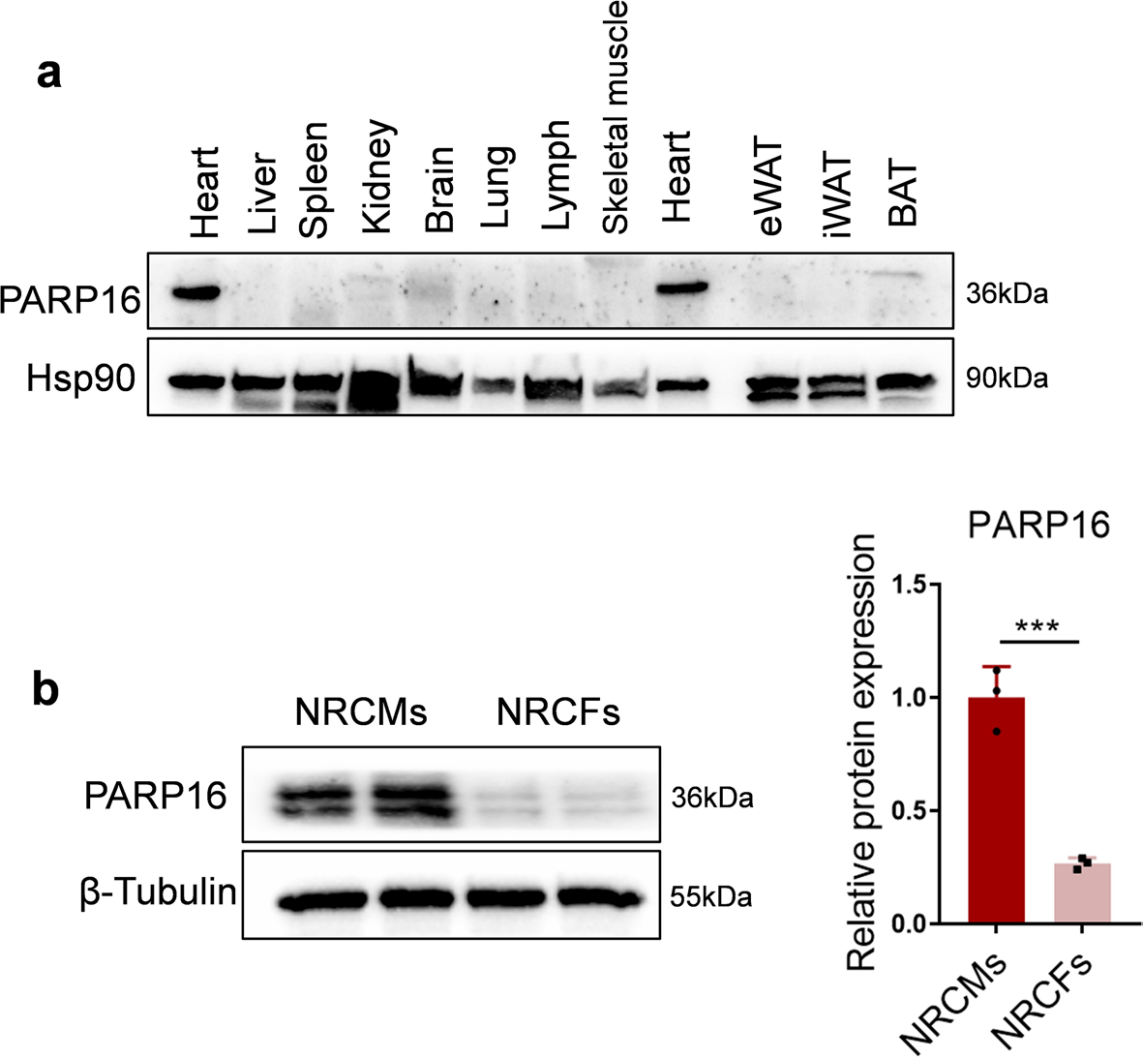
PARP16 expresses at high level in heart tissues and cardiomyocytes. **a** The high expression of PARP16 protein in the heart tissue. Eleven tissues including heart, liver, spleen, kidney, brain, lung, lymph, skeletal muscle and adipose tissue were collected from adult C57BL/6J mice and extracted proteins. Western blot was performed to examined the protein expression of PARP16 in each tissue. **b** The expression of PARP16 was compared in NRCMs and NRCFs separated from newborn rats by western blot analysis. Data are presented as mean ± SD, and analyzed using Student *t* test. *n* = 3, ***p < 0.001

Firstly, we examined the expression profile of PARP16 in different organs of mice. The results showed that PARP16 was highly expressed in the heart tissue (Fig. 1a). Furthermore, PARP16 mainly expressed in neonatal rat cardiomyocytes (NRCMs) but not the cardiac fibroblasts in the hearts of 1–2 days of SD rats (Fig. 1b), suggesting that PARP16 may play an underlying role in cardiovascular diseases.

### PARP16 increases in pathological cardiac hypertrophy both in vivo and in vitro

**Fig. 2:**
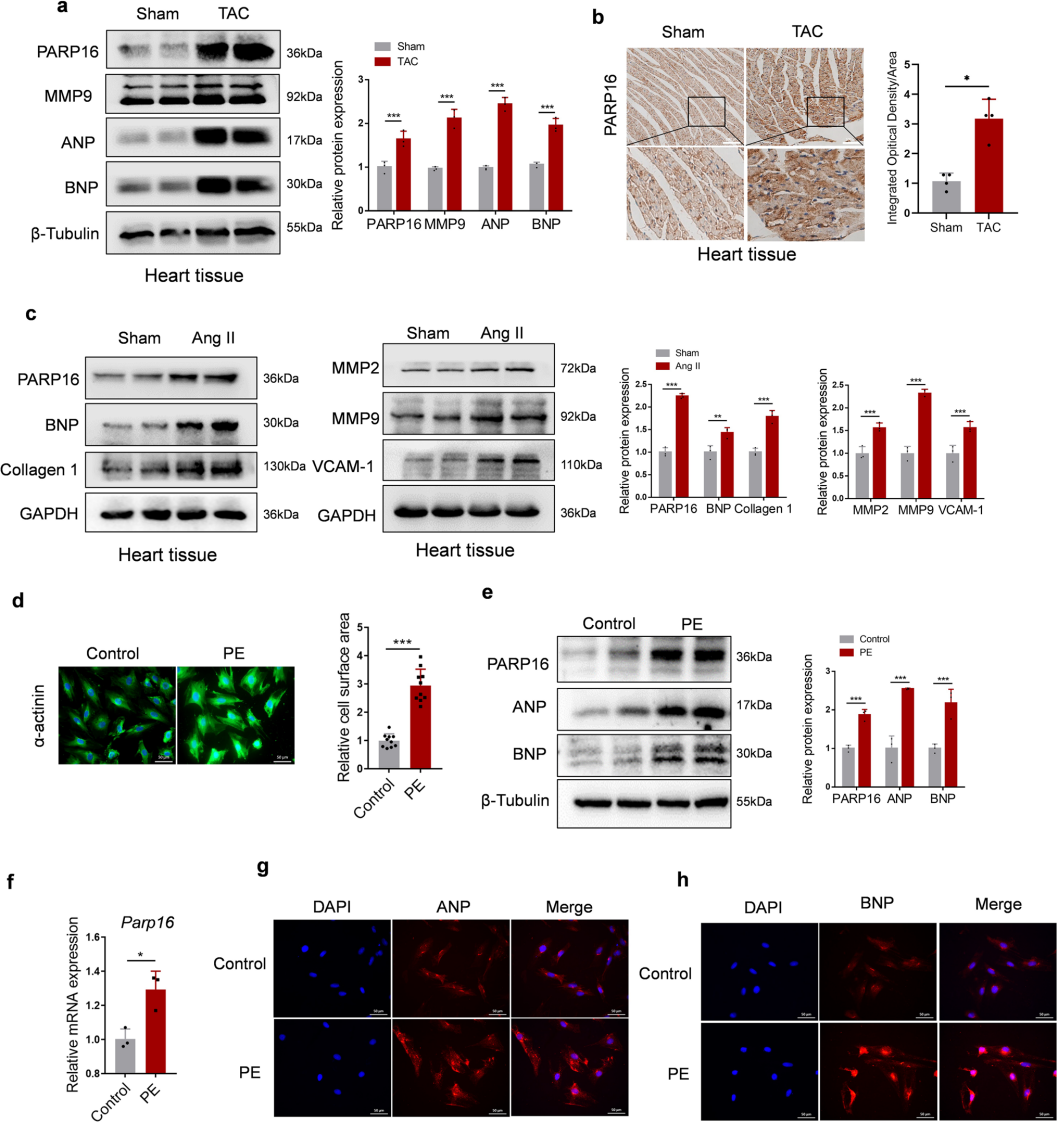
PARP16 increases in pathological cardiac hypertrophy both in vivo and in vitro*.* **a** The protein expressions of PARP16, fibrotic marker MMP9 and hypertrophic marker (ANP, BNP) in heart tissues from mice subjected to sham or TAC surgery were detected by western blot. **b** Representative images of immunohistochemistry staining with anti-PARP16 in heart sections from sham and TAC groups. *n* = 3/group. Scale bar: 100 μm. **c** The protein expressions of PARP16, the myocardial hypertrophy marker BNP, myocardial fibrotic markers (Collagen 1 and MMP2/9) and the inflammatory marker VCAM-1 in heart tissues from mice subjected to sham or Ang II infusion were detected by western blot. **d** The immunofluorescence staining with anti-α-actinin in PE (100 µM, 48 h)-induced H9C2 cells. Scale bar: 50 μm. Quantification of cell surface area as shown on the right. **e** The protein expressions of PARP16 and myocardial hypertrophic markers (ANP, BNP) in PE-induced H9C2 cells were detected by western blot. **f** The mRNA level of PARP16 in PE-treated H9C2 cells were detected by RT-qPCR. **g**, **h** Representative immunofluorescence staining images of ANP or BNP in PE-treated H9C2 cells. Scale bar: 50 μm. Data are presented as mean ± SD, and analyzed using Student *t* test or one-way ANOVA followed by Tukey Post-hoc tests. *n* = 3, *p < 0.05, **p < 0.01, ***p < 0.001

To elucidate the underlying role of PARP16 in pathological cardiac hypertrophy, transverse aortic constriction (TAC) surgery was performed to construct the experimental cardiac hypertrophy model in C57BL/6J male mice. As shown in Fig. S1a, the heart morphological phenotype and structure obviously enlarged at day 28 after TAC surgery compared with the sham group. Moreover, the cross-sectional areas of cardiomyocytes stained with WGA from the TAC-operated mice were significantly larger than those of sham mice (Fig. S1b). In addition, hypertrophic markers such as ANP and BNP, and the cardiac fibrotic marker MMP9 were also substantially increased in TAC-challenged hypertrophic hearts compared with sham group, suggesting that TAC-induced myocardial hypertrophy model was successfully constructed (Fig. 2a). Interestingly, the expression of PARP16 protein was profoundly augmented in TAC-challenged hypertrophic hearts as determined both by western blot and IHC assay (Fig. 2a, b). Taking it a step further, Angiotensin II (Ang II) is known to be a degrading peptide of the renin–angiotensin system and is one of the most powerful stimulants inducing cardiac hypertrophy and fibrosis. Mice continuously infused with Ang II were used as a classic model of cardiac hypertrophy [14]. In the present study, the protein expression of PARP16 was also increased in the heart tissue of Ang II-infused mice, accompanied with the upregulation of myocardial hypertrophy marker BNP, myocardial fibrotic markers (Collagen 1 and MMP2/9) and the inflammatory marker VCAM-1 (Fig. 2c).

To further investigate whether PARP16 was associated with pathological cardiac hypertrophy in vitro, H9C2 cells or neonatal rat cardiomyocytes (NRCMs) were treated with pathologic hypertrophy agonist phenylephrine (PE), an α-adrenergic stimulant. As shown in Fig. 2d, e and Fig. S2a, b, PE-induced cardiomyocytes hypertrophic growth was confirmed by dramatically increased cardiomyocytes area stained with α-actinin, as well as the upregulated protein expressions of ANP and BNP. In addition, the immunofluorescence staining of ANP or BNP showed the similar pattern (Fig. 2g, h). It is noteworthy that both protein and mRNA levels of PARP16 were distinctly upregulated in PE-treated H9C2 cells and NRCMs (Fig. 2e, f and Fig. S2b), implying that PARP16 may be correlated with the progress of pathological cardiac hypertrophy.

### Depletion of PARP16 alleviates PE-induced cardiac hypertrophy in vitro

**Fig. 3:**
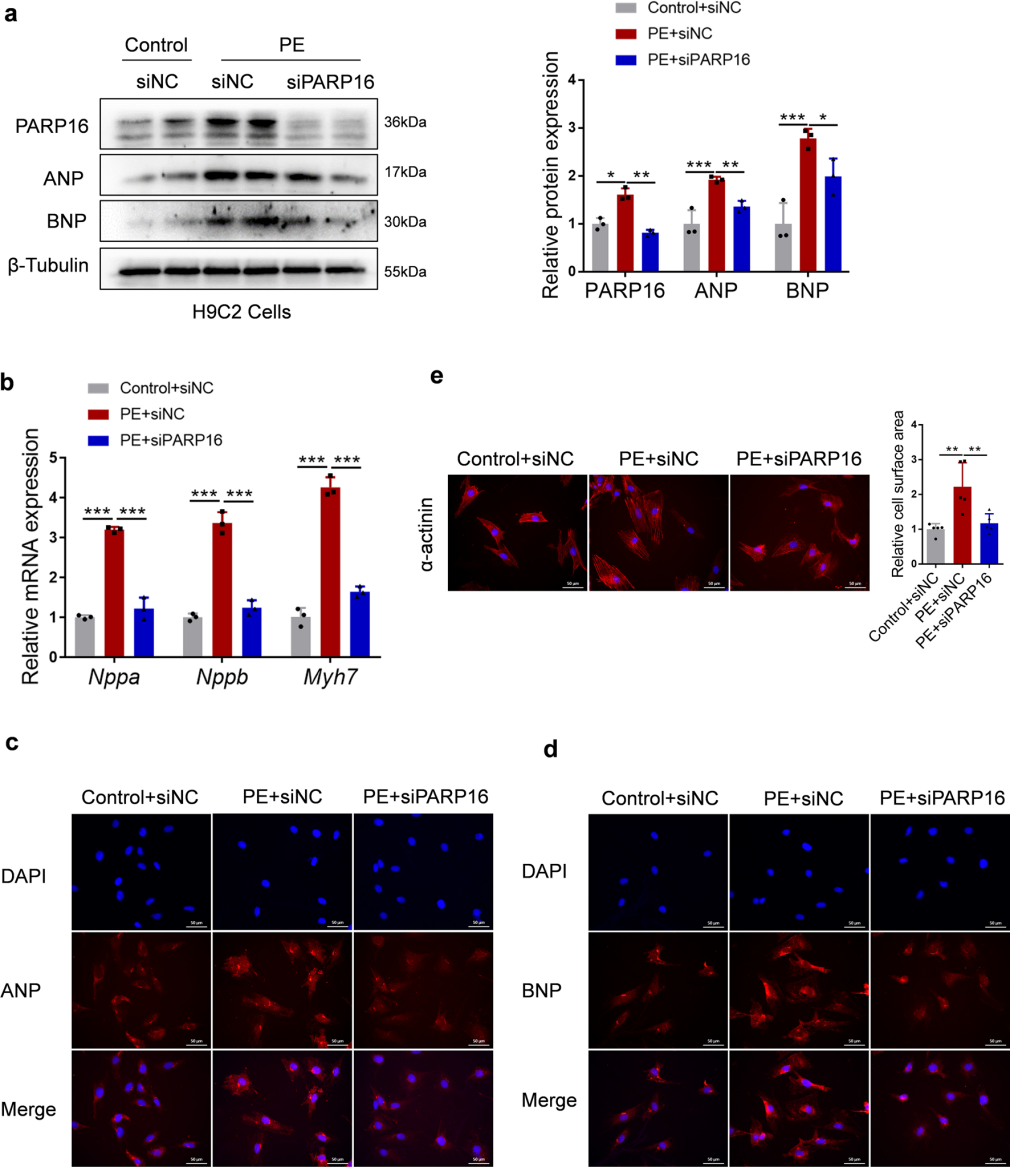
Depletion of PARP16 alleviates PE-induced cardiomyocytes hypertrophy in vitro*.* H9C2 cells were transfected with PARP16-specific short interfering RNA (siPARP16) and then treated with PE (100 µM, 48 h). **a** Representative western blot (left) and quantification (right) of PARP16, ANP and BNP expressions. **b** The mRNA levels of hypertrophic marker genes (*Nppa*, *Nppb*, *Myh7*) were detected by RT-qPCR analysis. **c**, **d** Representative immunofluorescence staining images of ANP or BNP. Scale bar: 50 μm. **e** Representative immunofluorescence images of α-actinin staining. Scale bar: 50 μm. Quantification of cell surface area as shown on the right. Data are presented as mean ± SD, and analyzed using one-way ANOVA followed by Tukey Post-hoc tests. *n* = 3, *p < 0.05, **p < 0.01, ***p < 0.001

In light of the association between PARP16 and pathological cardiac hypertrophy, we next set out to test for a causal role of PARP16 in this pathogenesis. Knockdown of PARP16 using siPARP16 was performed with the presence of hypertrophic stimulation PE in both H9C2 and NRCMs. As indicated in Fig. 3a, b and Fig. S2c, both protein and mRNA levels of hypertrophic markers ANP and BNP or *Myh7* mRNA level were potentiated upon PE treatment whereas markedly reduced by PARP16 silencing. Consistently, similar observations were obtained by the immunofluorescence staining of ANP or BNP in H9C2 cells (Fig. 3c, d). Furthermore, PE-induced increase of cardiomyocyte surface areas was also ameliorated by knockdown of endogenous PARP16 (Fig. 3e and Fig. S2d). These results demonstrate that PARP16 deficiency alleviates the progression of cardiomyocyte hypertrophic growth in vitro.

### PARP16 overexpression activates cardiomyocyte hypertrophic responses in vitro

**Fig. 4:**
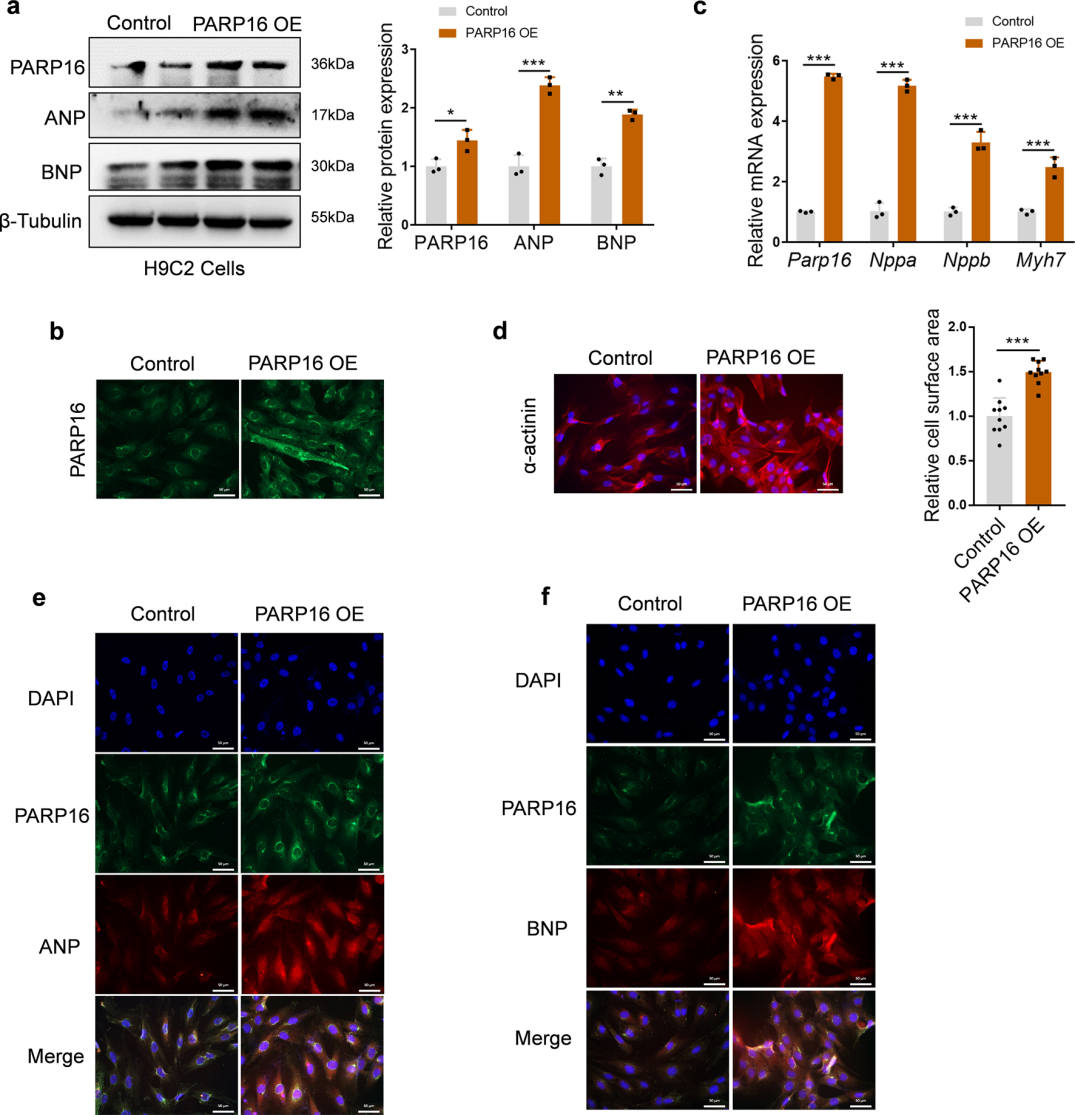
PARP16 overexpression activates hypertrophic responses in vitro. H9C2 cells were transfected with PARP16 OE lentivirus. **a** Representative western blots of PARP16, ANP and BNP. **b** Representative immunofluorescence images of PARP16 staining. Scale bar: 50 μm. **c** The mRNA levels of *Parp16* and hypertrophic marker genes (*Nppa*, *Nppb*, *Myh7*) were detected by RT-qPCR analysis. **d** Representative immunofluorescence images of α-actinin staining. Scale bar: 50 μm. Quantification of cell surface area as shown on the right. **e**, **f** Representative immunofluorescence double staining images of PARP16 and ANP or BNP. Scale bar: 50 μm. Data are presented as mean ± SD, and analyzed using Student *t* test. *n* = 3, *p < 0.05, **p < 0.01, ***p < 0.001

To further explore the regulatory role of PARP16 in cardiac hypertrophy, we overexpressed PARP16 by transfecting the PARP16 overexpression lentivirus (PARP16 OE) in both H9C2 cells and NRCMs. As shown in Fig. 4a, b, PARP16 OE enhanced the expression of PARP16 as determined by both western blot and immunofluorescence staining assays. Meanwhile, PARP16 OE directly triggered the hypertrophic responses, as indicated by the upregulation of ANP and BNP protein and mRNA levels or *Myh7* mRNA level (Fig. 4a, c and Fig. S2e), as well as the augmented cardiomyocyte surface areas (Fig. 4d and Fig. S2f). To further assess, we performed immunofluorescence double staining and found a higher co-expression of PARP16 and hypertrophic markers ANP or BNP (Fig. 4e, f). Taken together, these above observations indicate that PARP16 overexpression led to hypertrophic responses, suggesting PARP16 is required for the progression of cardiomyocyte hypertrophic growth in vitro.

### PARP16 deficiency ameliorates pathological cardiac hypertrophy in vivo

**Fig. 5:**
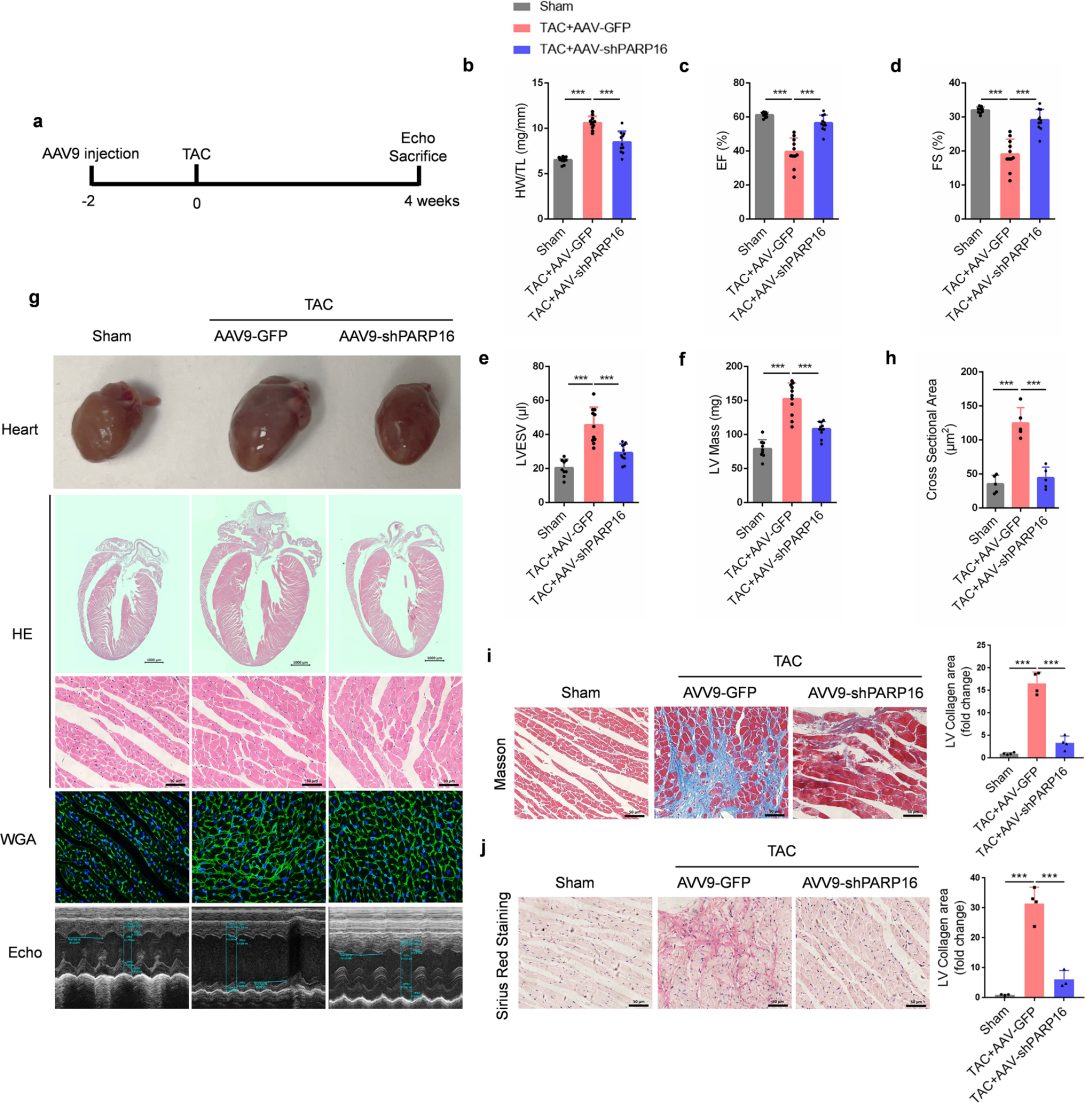
PARP16 deficiency ameliorates pathological cardiac hypertrophy in vivo*.* Adeno-associated viruses AAV9-PARP16 shRNA (AAV9-shPARP16) were injected into the left ventricle of mice for 4–5 dots to drive the knockdown of PARP16, then followed by TAC surgery for 4 weeks, AAV9-GFP was used as the negative control. **a** The schematic diagram depicting the design of the animal study. **b** The heart weight/tibia length (HW/TL) ratios. *n* = 9 or 11/group. **c** The ejection fraction (EF%). *n* = 9 or 11/group. **d** The fractional shortening (FS%). *n* = 9 or 11/group. **e** LVESV (LV volume of systole), *n* = 9 or 11/group. **f** LV mass. *n* = 9 or 11/group. **g** Representative images of heart sections by the gross morphologic examination, hematoxylin and eosin (H&E) staining, wheat germ agglutinin (WGA) staining as well as echocardiography images results from WT mice injected with AAV9-GFP or AAV9-shPARP16 subsequently challenged to sham or TAC surgery. **h** The quantitative cross-sectional diameter of cardiomyocytes by WGA staining. *n* = 5/group. **i**, **j** Histological analysis of the Masson staining (**i**) and Sirius Red Staining (**j**) of heart sections from WT mice injected with AAV9-GFP or AAV9-shPARP16 subsequently challenged to sham or TAC surgery. The quantification results of LV interstitial collagen volume were on the right. *n* = 3 or 4/group. Data are presented as mean ± SD and analyzed using one-way ANOVA followed by Tukey Post-hoc tests. ***p < 0.001

Having validated the finding that PARP16 successfully regulated the cardiomyocytes hypertrophic response in vitro, we next sought to determine the impact of PARP16 on TAC-induced cardiac hypertrophy in vivo. Here, AAV9-shPARP16 was injected into the left ventricle of mice for 4–5 dots to drive the knockdown of PARP16, AAV9-GFP was used as the negative control. Two weeks later, these mice were then challenged by TAC surgery for another 4 weeks (Fig. 5a).

Firstly, we examined the transfection efficiency of AAV9 by detecting the green fluorescence after intramyocardial injection of AAV9-GFP and found that green fluorescence was throughout the heart from the left ventricle to the apex (Fig. S3). Next, we detected whether PARP16 ablation alleviated TAC-induced cardiac hypertrophy. As shown in Fig. 5b–g, knockdown of PARP16 by AAV9-shPARP16 inhibited TAC-induced heart weight/tibia length (HW/TL) ratios (Fig. 5b) and improved the cardiac function (Fig. 5g, the last panel), which was also indicated by upregulation of left ventricular (LV) ejection fraction (EF %) (Fig. 5c) and LV fractional shortening (FS %) (Fig. 5d) and downregulation of LV end-systolic volume (LVESV) and LV mass (Fig. 5e, f). Meanwhile, other echocardiographic parameters representing cardiac function such as IVSd, IVSs and LV internal dimension at systole (LVID; s) were also listed in the Table S2. Consistent with these findings, PARP16 deficiency also decreased the heart size by gross morphologic examination and H&E staining, as well as reduced the cross-sectional diameter of cardiomyocytes by WGA staining (Fig. 5g, h). Moreover, we examined interstitial fibrosis, another hypertrophic pathological phenotype, and found PARP16 deficiency notably dampened interstitial fibrosis in TAC-induced experimental cardiac hypertrophy model by Masson and Sirius Red Staining of heart sections **(**Fig. 5i, j).

**Fig. 6:**
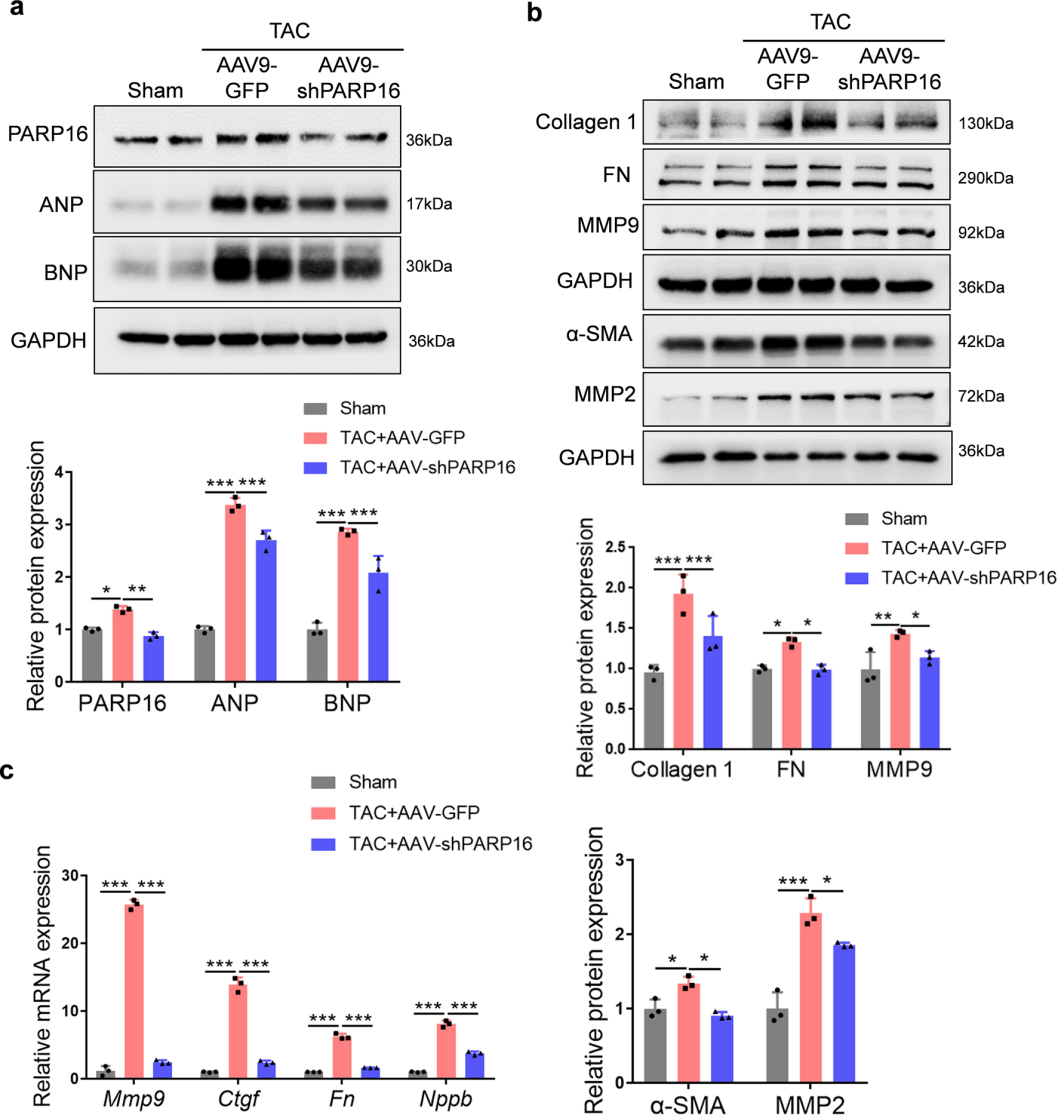
Ablation of PARP16 downregulated the hypertrophic and fibrotic marker expressions. **a**, **b** The protein expressions of hypertrophic markers (ANP, BNP) and fibrotic markers (Collagen 1, FN, MMP2/9 and α-SMA) in heart sections from WT mice injected with AAV9-GFP or AAV9-shPARP16 subsequently subjected to sham or TAC surgery by immunoblot analysis. The quantification results were listed below. *n* = 3. **c** The mRNA levels of fibrotic marker gene (*Fn, Mmp9 *and* Ctgf*) and hypertrophic marker gene (*Nppb*) in heart sections from WT mice injected with AAV9-GFP or AAV9-shPARP16 subsequently subjected to sham or TAC surgery by RT-qPCR analysis. *n* = 3. Data are presented as mean ± SD and analyzed using one-way ANOVA followed by Tukey Post-hoc tests. *p < 0.05, **p < 0.01, ***p < 0.001

Taking it a step further, consistent with the in vitro observations, the in vivo results also confirmed that the hypertrophic-related protein (ANP and BNP) and fibrotic-related protein (Collagen1, FN, MMP2/9 and α-SMA) expressions were increased in TAC-induced cardiac hypertrophy but reduced by PARP16 knockdown (Fig. 6a, b). Moreover, the upregulated hypertrophic marker gene *Nppb* (encoding BNP) and fibrotic genes (*Fn*, *Mmp9* and *Ctgf*) by TAC operation were diminished by PARP16 knockdown (Fig. 6c). To further assess, PARP16 ablation also decreased the expression of myocardial fibrotic markers (MMP9 and α-SMA) in cardiac tissues from Ang II-infusion mice with PARP16 shRNA lentivirus infection for 4 weeks (Fig. S4). Collectively, these above results elucidated that PARP16 deficiency by gene delivery via AAV9 vector or lentivirus infection could ameliorate TAC- or Ang II infusion-induced cardiac hypertrophy or fibrosis and improve the cardiac function in vivo*.*

### PARP16 regulated the IRE1α–sXBP1 pathway in response to the hypertrophic stimuli

**Fig. 7:**
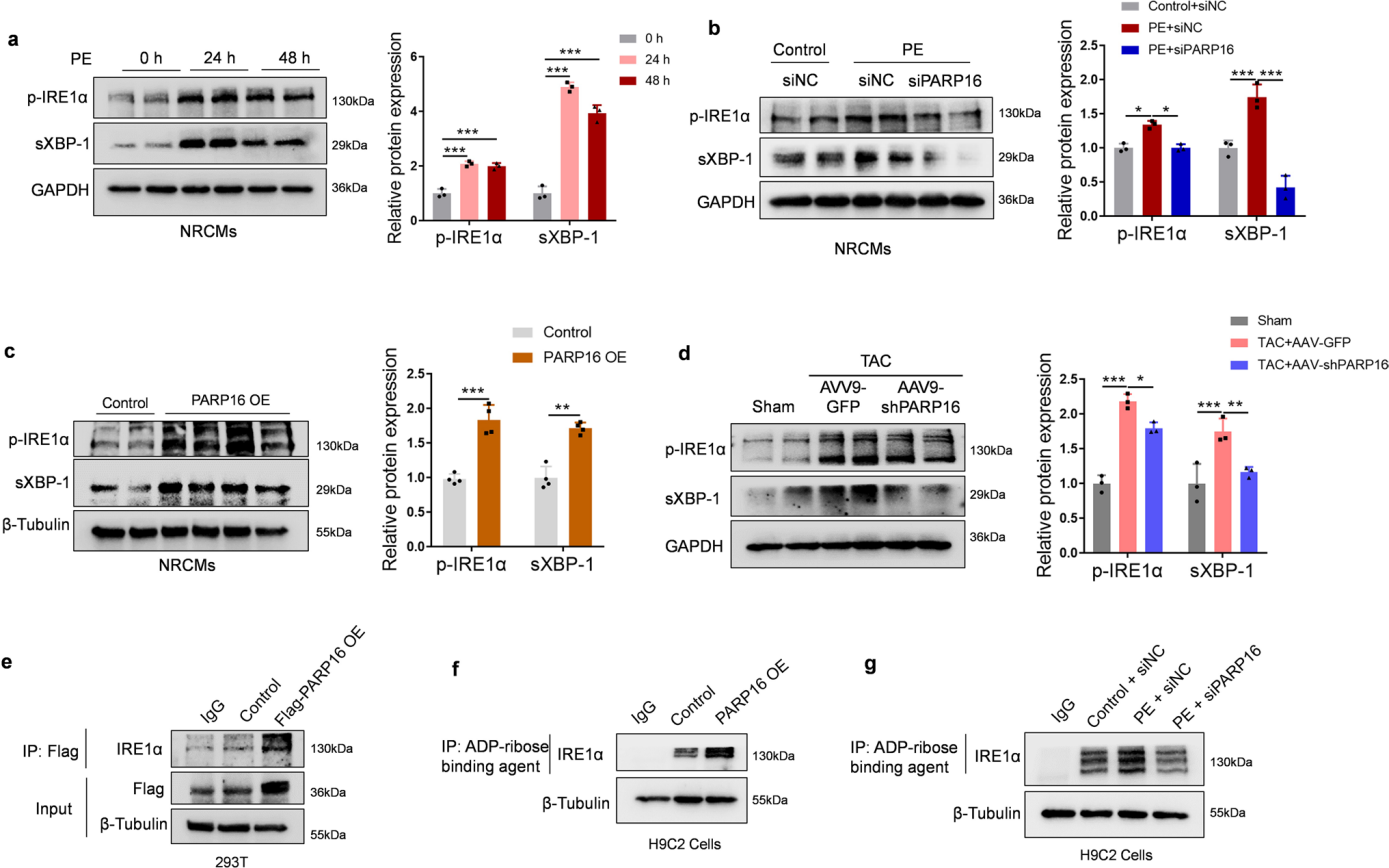
PARP16 regulated the IRE1α–sXBP1 pathway in response to the hypertrophic stimuli. **a** The activation of IRE1α–sXBP1 pathway in PE-treated NRCMs. **b** PARP16 knockdown abolished the activation of IRE1α–sXBP1 pathway by western blot assay. **c** PARP16 OE directly activated the IRE1α–sXBP1 pathway in NRCMs. **d** The IRE1α–sXBP1 pathway was also regulated in heart sections from WT mice injected with AAV9-GFP or AAV9-shPARP16 subsequently subjected to sham or TAC surgery by immunoblot analysis. **e** The interaction between PARP16 and IRE1α was determined by co-IP assay in 293T cells transfected by Flag-tagged PARP16 overexpressed lentiviruses. **f**, **g** The ADP-ribosylation level of IRE1α was detected by co-IP assay using the ADP-ribose binding agent. Data are presented as mean ± SD and analyzed using Student *t* test or one-way ANOVA followed by Tukey Post-hoc tests. *n* = 3. *p < 0.05, **p < 0.01, ***p < 0.001

Given that UPR is involved in the pathogenesis of cardiac hypertrophy [15] as well as PARP16 is correlated with the activation of the UPR sensors PERK and IRE1α [8], we examined the expressions of the PERK and IRE1α signaling in PE-treated NRCMs or H9C2 cells. Western blot analysis showed that both p-IRE1α and sXBP1 was significantly upregulated in PE-treated NRCMs (Fig. 7a), but the p-PERK-p-eIF2α pathway was hardly activated upon PE treatment (Fig. S5). Therefore, we focused on the effect of PARP16 on IRE1α–sXBP1 pathway. As seen in Fig. 7b, c, knockdown of PARP16 abolished while PARP16 OE directly triggered the activation of IRE1α–sXBP1 pathway. Moreover, the association between PARP16 and activated IRE1α–sXBP1 signaling was also verified in cardiac tissues from mice that injected with AAV9-shPARP16 and subsequently subjected to TAC surgery (Fig. 7d), suggesting that PARP16 modulates the IRE1α–sXBP1 pathway in TAC-induced cardiac hypertrophy.

Next, we aimed to determine whether PARP16 activated the IRE1α–sXBP1 pathway through directly interacting with the UPR sensors IRE1α. We constructed a Flag-tagged PARP16 overexpressed lentiviruses and transfected 293T cells as previously reported [11]. The cell lysates were collected for co-IP assay and the results showed that IRE1α was examined in precipitations pulled down by Flag-tagged antibody upon PARP16 overexpression (Fig. 7e), which provides the evidence of the interaction between PARP16 and IRE1α. Taking it a step further, as known that PARP16 was required for ADP-ribosylation modification of IRE1α during the UPR [8], we determined the ADP-ribosylation of IRE1α in both PARP16 overexpressed- and PE-treated H9C2 cells. Co-IP assay using the ADP-ribose binding agent were performed to investigate the ADP-ribosylation level of IRE1α. As indicated in Fig. 7f, g, PARP16 OE or PE treatment increased while knockdown of PARP16 blunted the ADP-ribosylation level of IRE1α. These above results demonstrated that PARP16 selectively interacted with IRE1α and ADP-ribosylated IRE1α in response to the hypertrophic stimuli.

### PARP16 activated GATA4 via IRE1α–sXBP1 pathway in cardiac hypertrophy

**Fig. 8:**
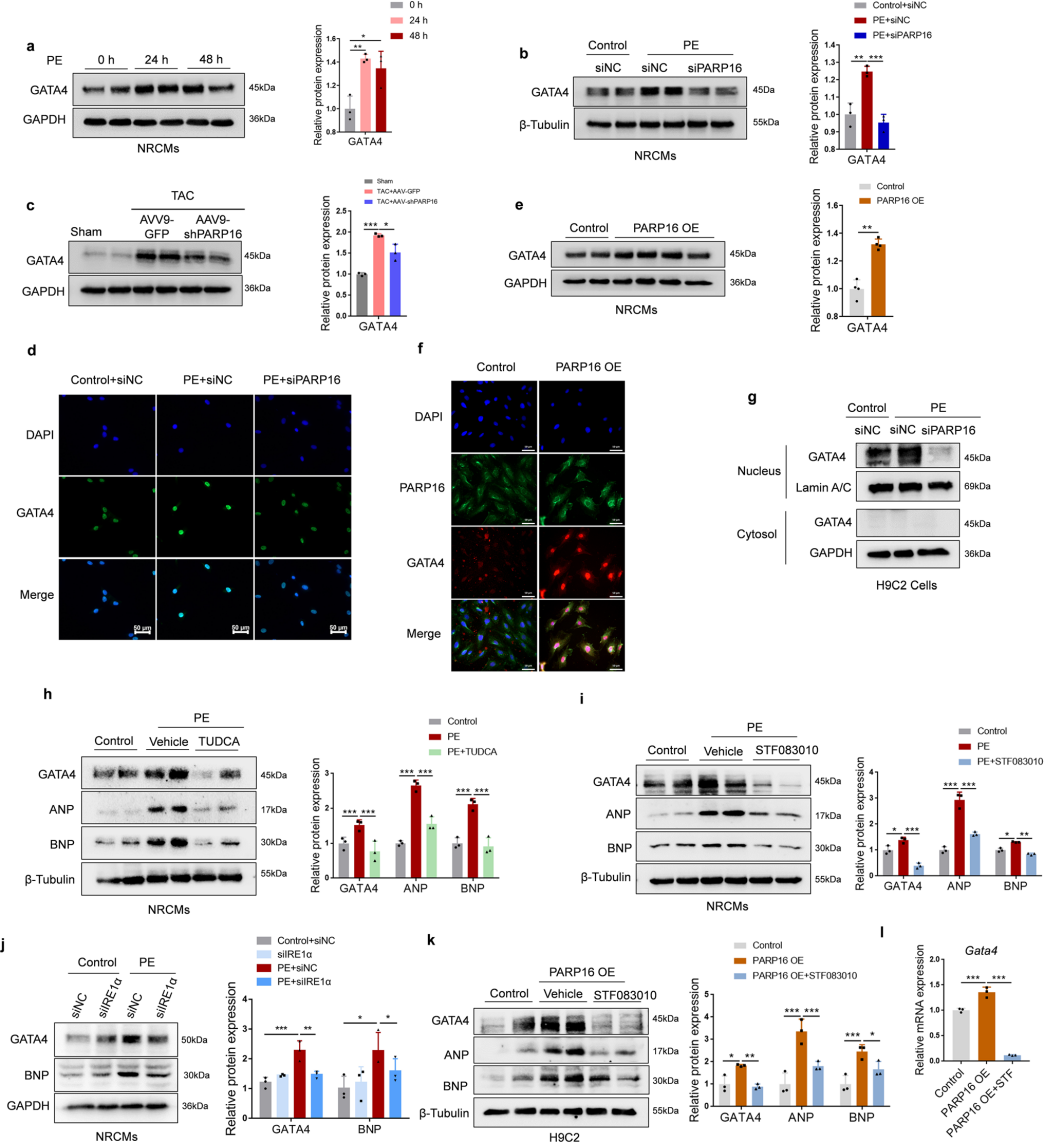
PARP16 activated GATA4 via IRE1α–sXBP1 pathway in cardiac hypertrophy. **a** The protein expression of GATA4 in PE-treated NRCMs. **b** Knockdown of PARP16 abolished the activation of GATA4 in PE-treated NRCMs by western blot assay. **c** The protein expression of GATA4 was also regulated in heart sections from WT mice injected with AAV9-GFP or AAV9-shPARP16 and subsequently subjected to sham or TAC surgery by immunoblot analysis. **d** Representative immunofluorescence staining images of GATA4 in PARP16 knockdown followed by PE treatment. scale bar: 50 μm. **e** PARP16 OE directly activated the expression of GATA4 in NRCMs. **f** Representative immunofluorescence double staining images of PARP16 and GATA4 in PARP16 OE-transfected H9C2 cells. Scale bar: 50 μm. **g** The effects of PARP16 knockdown on the nuclear and cytoplasm distribution and expression of GATA4 by western blot. **h**, **i** The effects of the ER stress inhibitor (TUDCA) and the specific IRE1α inhibitor STF-083010 on the expressions of GATA4 as well as the hypertrophic markers ANP and BNP in response to PE treatment. **j** Knockdown of IRE1α abolished the activation of GATA4 or hypertrophic marker BNP in PE-treated NRCMs. **k** The effects of the specific IRE1α inhibitor STF-083010 on the protein expression of GATA4 as well as the hypertrophic marker ANP and BNP upon PARP16 OE treatment. **l** The effects of STF-083010 on the mRNA level of GATA4 upon PARP16 OE treatment. Data are presented as mean ± SD and analyzed using Student *t* test or one-way ANOVA followed by Tukey Post-hoc tests. *n* = 3. *p < 0.05, **p < 0.01, ***p < 0.001

Studies have also documented that the transcription factor GATA4 is pivotal to regulate the expression of cardiac-specific genes that promote cardiomyocyte hypertrophy [16]. Furthermore, cardiomyocyte-specific GATA4-deficient or transgenic mice has revealed its requirement for the development of cardiac hypertrophy [17, 18]. Therefore, we were curious to determine whether PARP16 regulates GATA4 expression in response to TAC surgery or PE treatment. Here we found that GATA4 was significantly increased in both PE-treated NRCMs and TAC-operated heart tissue while downregulated by PARP16 deficiency (Fig. 8a–c). Similar patterns were observed with the immunofluorescence staining result (Fig. 8d). Furthermore, GATA4 was also activated directly by PARP16 OE, as evidenced by the high protein level and immunofluorescence intensity (Fig. 8e, f). To further confirm the influence of PARP16 on GATA4 localization, nuclear and cytoplasmic proteins were extracted and found PARP16 knockdown markedly decreased the expression of GATA4 in nucleus but not cytoplasm upon PE treatment (Fig. 8g). Collectively, these above results suggested that PARP16 regulates the expression of GATA4 in response to the cardiomyocyte hypertrophic growth.

Interestingly, we have uncovered that PARP16 is an ER transmembrane protein that could interact with IRE1α and activated the IRE1α–sXBP1 pathway during PE-stimulated hypertrophic growth, and GATA4 was a transcription factor primarily located in the nucleus [19], also as evidenced in Fig. 8d, f, g. These outcomes led us to hypothesize that PARP16 may not directly mediate GATA4 activation and maybe through the IRE1α–sXBP1 pathway. To test this hypothesis, both tauroursodeoxycholic acid (TUDCA) (an ER stress inhibitor) and STF-083010 (a specific IRE1α inhibitor) were used to examine the relationship between IRE1α–sXBP1 pathway and GATA4. Indeed, treatment with either TUDCA or STF-083010 could blunt the expressions of GATA4 as well as the hypertrophic marker ANP and BNP in response to PE treatment (Fig. 8h, i). In addition, silencing IRE1α through siRNA upon PE stimulation decreased the protein expressions of GATA4 as well as the hypertrophic marker BNP in NRCMs (Fig. 8j). Importantly, the specific IRE1α inhibitor STF-083010 also attenuated PARP16 OE-induced upregulation of GATA4 both in protein and mRNA levels (Fig. 8k, l), suggesting that PARP16 mediated GATA4 upregulation maybe at least in part via the IRE1α–sXBP1 pathway.

### The expression of PARP16 is mediated by JNK/ERK MAPK pathway in PE-induced cardiomyocyte hypertrophy

To further identify the upstream regulatory pathway of PE-induced PARP16 upregulation, we examined mitogen-activated protein kinase (MAPK) signaling pathway, a classical pathway involved in cardiac hypertrophy [20]. Our results showed that the phosphorylation of MAPK pathway members, including JNK, ERK1/2, except p38 MAPK were increased upon PE challenge in vitro (Fig. S6a)*.* Then the specific inhibitors of JNK, ERK1/2 and p38 MAPK, namely SP600125, PD98059 and SB203580 were used and found the expression of PARP16 was inhibited by JNK and ERK1/2 inhibitors but not p38 MAPK inhibitors (Fig. S6b), suggesting that PE-induced PARP16 upregulation maybe mediated by JNK/ERK MAPK pathway.

## Discussion

Pathological cardiac hypertrophy is regarded as an independent risk factor of HF, whose current main therapeutic interventions are still a bottleneck problem [1]. Thus, a deeper understanding of the mechanisms involved in pathological cardiac hypertrophy is necessary for exploring the potential drug targets. In the present study, we determined that PARP16 was a key driver of pathological cardiac hypertrophy. PARP16 deficiency rescued cardiac dysfunction and ameliorated TAC-induced cardiac hypertrophy and fibrosis in mice, as well as PE-induced cardiomyocyte hypertrophic responses. Whereas overexpression of PARP16 exacerbated the hypertrophic responses. Mechanistically, PARP16 interacted with IRE1α and ADP-ribosylated IRE1α and mediated the hypertrophic responses through activating the IRE1α–sXBP1–GATA4 pathway. Collectively, our results implicated that PARP16 may be a contributor of pathological cardiac hypertrophy at least partly via activating the IRE1α–sXBP1–GATA4 pathway, and may be regarded as a new potential target for exploring effective therapeutic interventions of pathological cardiac hypertrophy and HF.

The poly (ADP-ribose) polymerases (PARPs) family members such as PARP-1 and PARP-2 has been revealed to participate in the progression of cardiac hypertrophy due to different cellular localization and regulating mechanisms. PARP-1 contributes to caspase-independent myocyte cell death during heart failure [21] while knockdown of PARP-2 protects against cardiac hypertrophy via SIRT1 activation [22]. However, PARP16 is the only PARP family member to be located on the endoplasmic reticulum and its role in cardiovascular diseases remains to be clarified. Recently, emerging evidence has demonstrated that PARP16 is identified as a novel target for vascular diseases, such as vascular aging and neointimal hyperplasia related diseases [11, 12]. Consistent with this, we were curious about the effect of PARP16 on pathological cardiac hypertrophy and observed PARP16 was at a higher level in response to pressure overload-induced pathological cardiac hypertrophy. PARP16 deficiency rescued cardiac dysfunction and ameliorated TAC-induced cardiac hypertrophy and fibrosis as well as PE-induced cardiomyocyte hypertrophic responses. Whereas overexpression of PARP16 exacerbated the hypertrophic responses, implicating that PARP16 may be a contributor of pathological cardiac hypertrophy.

It is also well documented that GATA transcription factors have important roles to promote ER integrity and Gata4 and Gata6 knockout mice could lead to several ER stress signals activation, such as CHOP [23]. However, the relationship between ER stress and GATA4 hasn't been explained well in pathological cardiac hypertrophy. In our study, we provided evidence that PARP16 could regulate the transcription expression of GATA4 to some extent depending on the IRE1α–sXBP1 pathway by using two molecular inhibitors (TUDCA, an ER stress inhibitor and STF-083010, a specific IRE1α inhibitor) as well as IRE1α siRNA. Indeed, treatment with either TUDCA or STF-083010 could blunt the expression of GATA4 in response to PE or PARP16 OE treatment. Furthermore, knockdown of IRE1α with siRNA significantly decreased the protein expression of GATA4 and BNP in PE-induced NRCMs, suggesting that PARP16 mediated expression of GATA4 at least in part via the IRE1α–sXBP1 pathway. Moreover, GATA4 acts as a key zinc finger-containing transcription factor of numerous cardiac-specific genes including *Nppa* and *Nppb* [16, 24] and ablation of GATA4 by siRNAs decreased ANP expression [25], therefore PARP16 contribute to the upregulation of ANP and BNP at least partly via activating the IRE1α–sXBP1–GATA4 pathway, further promote the progress of cardiac hypertrophy.

Due to the limited clinic resources, further verifications were not carried out in human samples, which is the deficiency of this study. Another limitation of this study are that we did not construct cardiac-specific knockout mice or cardiac-specific targeting adenoviruses, which should be explored in future studies.

## Supplementary Information

Below is the link to the electronic supplementary material.

Supplementary file1 (DOCX 15111 KB)

## Data Availability

All data generated or analyzed during this study are included in this published article and its supplementary information files.

## Abbreviations

AAV: Adeno-associated virus
AAV9-shPARP16: Serotype 9 AAV vectors encoding mouse PARP16 shRNA
ACEIs: Angiotensin converting enzyme inhibitors
Ang II: Angiotensin II
ATF6: Activating transcription factor 6
BrdU: Bromodeoxyuridine
Co-IP: Co-immunoprecipitation
DAPI: 4, 6-Diamidino-2-phenylindole
EF: Ejection fraction
EGCG: Epigallocatechin-3-gallate
FBS: Fetal bovine serum
FS: Fractional shortening
H&E: Hematoxylin and eosin
H3K4: Histone H3 lysine 4
HF: Heart failure
HW/TL: Heart weight/tibia length
IHC: Immunohistochemistry
IRE1α: Inositol requiring enzyme 1α
IVSd: The interventricular septal thickness at diastole
IVSs: The interventricular septal thickness at systole
LV: Left ventricular
LVESV: LV end-systolic volume
LVID; s: LV internal dimension at systole
MAPK: Mitogen-activated protein kinase
NC: Nitrocellulose
NRCMs: Neonatal rat cardiomyocytes
PARP: Poly (ADP-ribose) polymerase
PARP16 OE: PARP16 overexpression lentivirus
PE: Phenylephrine
PERK: Protein kinase R-like ER kinase
RT: Room temperature
RT-qPCR: Real-time and quantitative PCR
SD: Sprague–Dawley
shCTL: Lentivirus scramble shRNA
shPARP16: Lentivirus PARP16 shRNA
siIRE1α: IRE1α-specific short interfering RNA
siPARP16: PARP16-specific short interfering RNA
sXBP1: Spliced XBP1
TAC: Transverse aortic construction
TUDCA: Tauroursodeoxycholic acid
UPR: Unfolded protein response
uXBP1: Un-spliced XBP1
WGA: Wheat germ agglutinin
