# Supplementary material for: Poly (ADP-ribose) polymerases 16 triggers pathological cardiac hypertrophy via activating IRE1α–sXBP1–GATA4 pathway
Source: Cell Mol Life Sci. 2023 May 23;80(6):161. doi: 10.1007/s00018-023-04805-9 (PMC10205867; doi:10.1007/s00018-023-04805-9)
Supplement: Supplementary file 1 — Supplementary file1 (DOCX 15111 KB) [file 18_2023_4805_MOESM1_ESM.docx]

**SUPPLEMENTAL MATERIAL**

**Poly (ADP-ribose) polymerases 16 triggers pathological cardiac hypertrophy via activating IRE1α-sXBP1-GATA4 pathway**

**Running Title：***PARP16 Promotes Pathological Cardiac Hypertrophy*

Haibi Su ^1^, Jie Xu ^1^, Zhenghua Su ^1^, Chenxi Xiao ^1^, Jinghuan Wang ^1^, Wen Zhong ^1^, Chen Meng ^1^, Di Yang ^1,*^, Yizhun Zhu ^2,*^

*^1^School of Pharmacy, Pharmacophenomics Laboratory, Human Phenome Institute, Zhangjiang Fudan International Innovation Center, Fudan University, Shanghai, 201203 P.R. China*

*^2^State Key Laboratory of Quality Research in Chinese Medicine and School of Pharmacy, Macau University of Science and Technology, Macau, China*

**Address for Correspondence:**

Yizhun Zhu, PhD

State Key Laboratory of Quality Research in Chinese Medicine and School of Pharmacy, Macau University of Science and Technology, Avenida WaiLong, Taipa, Macau, China.

E-mail: yzzhu@must.edu.mo

Di Yang, PhD

Pharmacophenomics Laboratory, Human Phenome Institute, Fudan University

825, Zhangheng Road, Pudong New District, Shanghai, China.

E-mail: diyang@fudan.edu.cn


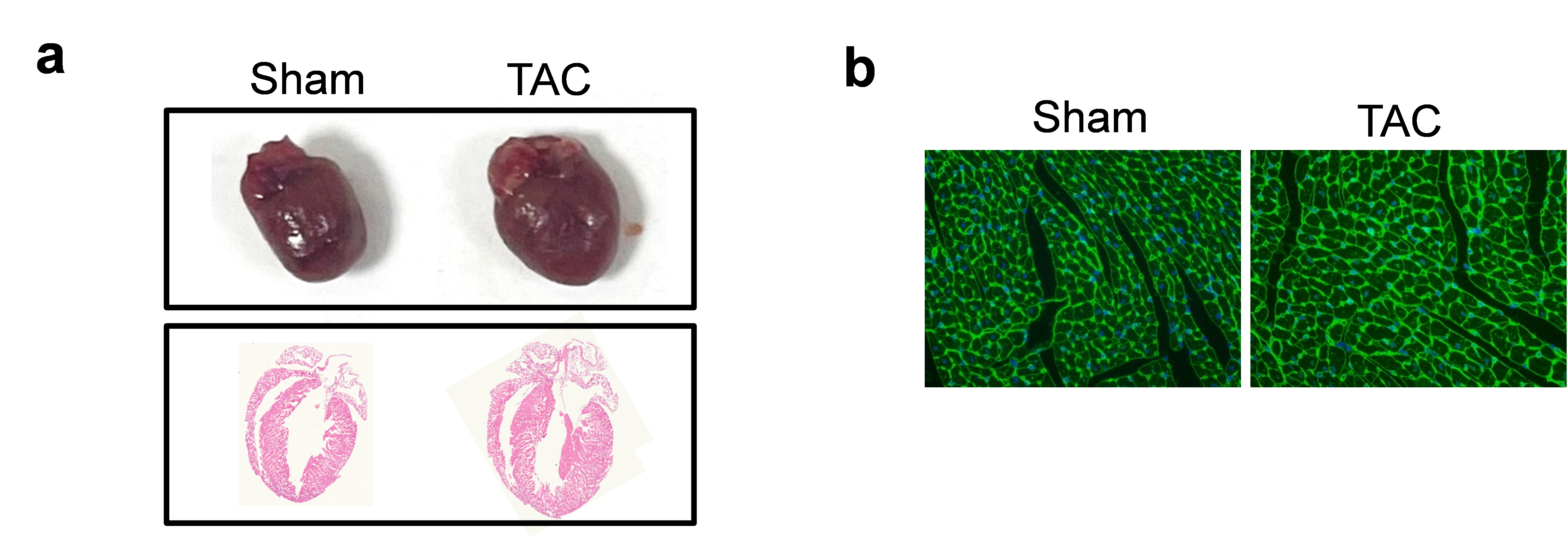


**Fig. S1 The experimental cardiac hypertrophy model is successfully constructed by transverse aortic constriction (TAC) surgery.** (**a**) Representative images of hearts from mice subjected to sham or TAC surgery by gross morphologic examination and H&E staining. (**b**) The WGA (wheat germ agglutinin) staining of hearts in sham and TAC group.


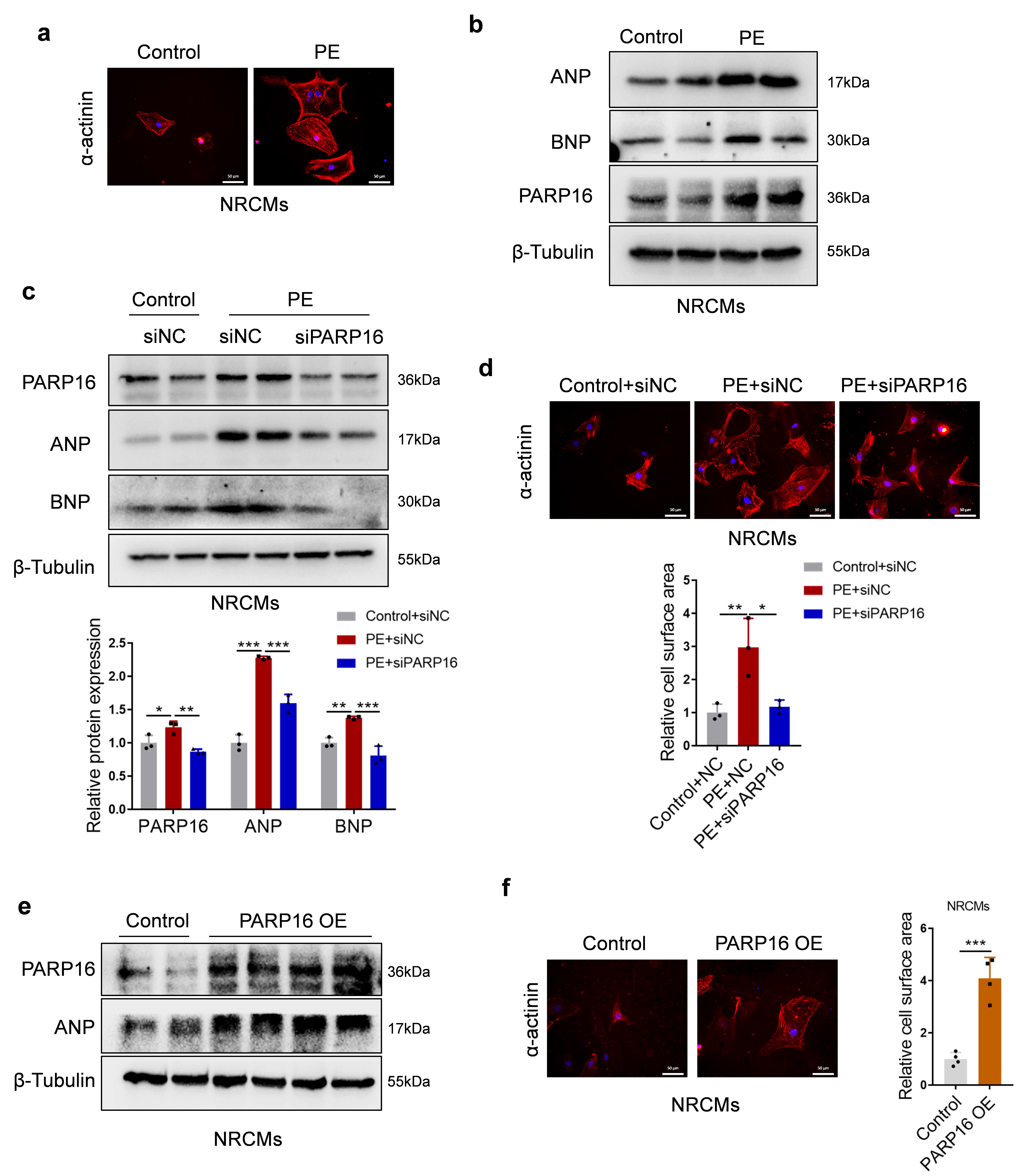


**Fig. S2 PARP16 regulates cardiomyocyte hypertrophy in NRCMs.** (**a**) The immunofluorescence staining with anti-α-actinin in PE (100 µM, 48h)-induced NRCMs cells. Scale bar: 50 μm. (**b**) The protein expressions of PARP16 and myocardial hypertrophic markers (ANP, BNP) in PE-induced NRCMs by western blot. (**c**) Representative western blot and quantification (below) of PARP16, ANP and BNP expressions in PE-treated NRCMs with or without PARP16 siRNA transfection. (**d**) Representative immunofluorescence images of α-actinin staining in PE-treated NRCMs with or without PARP16 siRNA transfection. Scale bar: 50 μm. Quantification of cell surface area is shown below. (**e-f**) Representative western blot of PARP16 and ANP and representative immunofluorescence images of α-actinin staining in PARP16 OE-transfected NRCMs. Scale bar: 50 μm. Quantification of cell surface area as shown on the right. Data are presented as mean ± SD, and analyzed using Student *t test* or one-way ANOVA followed by Tukey Post-hoc tests. n = 3. **p* < 0.05, ***p* < 0.01, ****p* < 0.001.


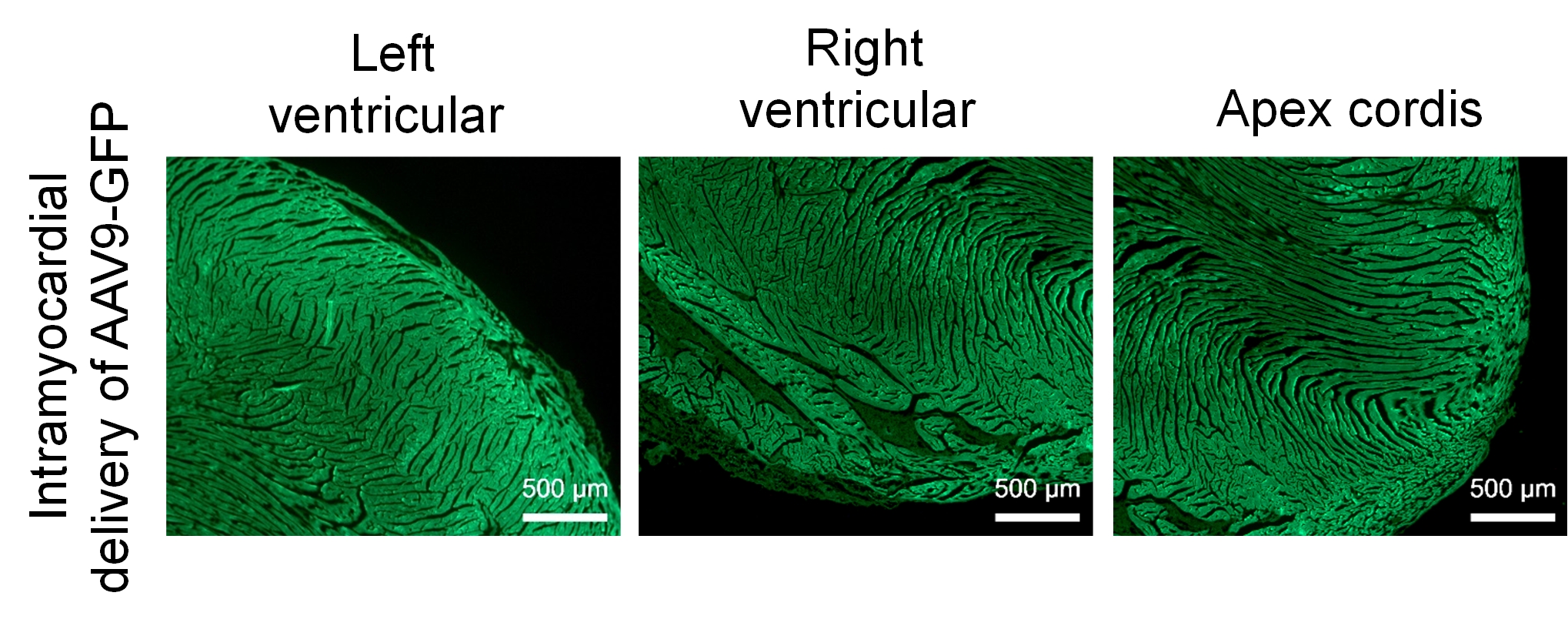


**Fig. S3 Verification of the delivery efficiency of AAV9-GFP via** **intramyocardial delivery.** The delivery efficiency of AAV9-GFP was verified by immunofluorescence staining in the myocardium of mice by showing that green fluorescence was all over the heart, from the left ventricle to the apex. Scale bar: 500 μm.

**
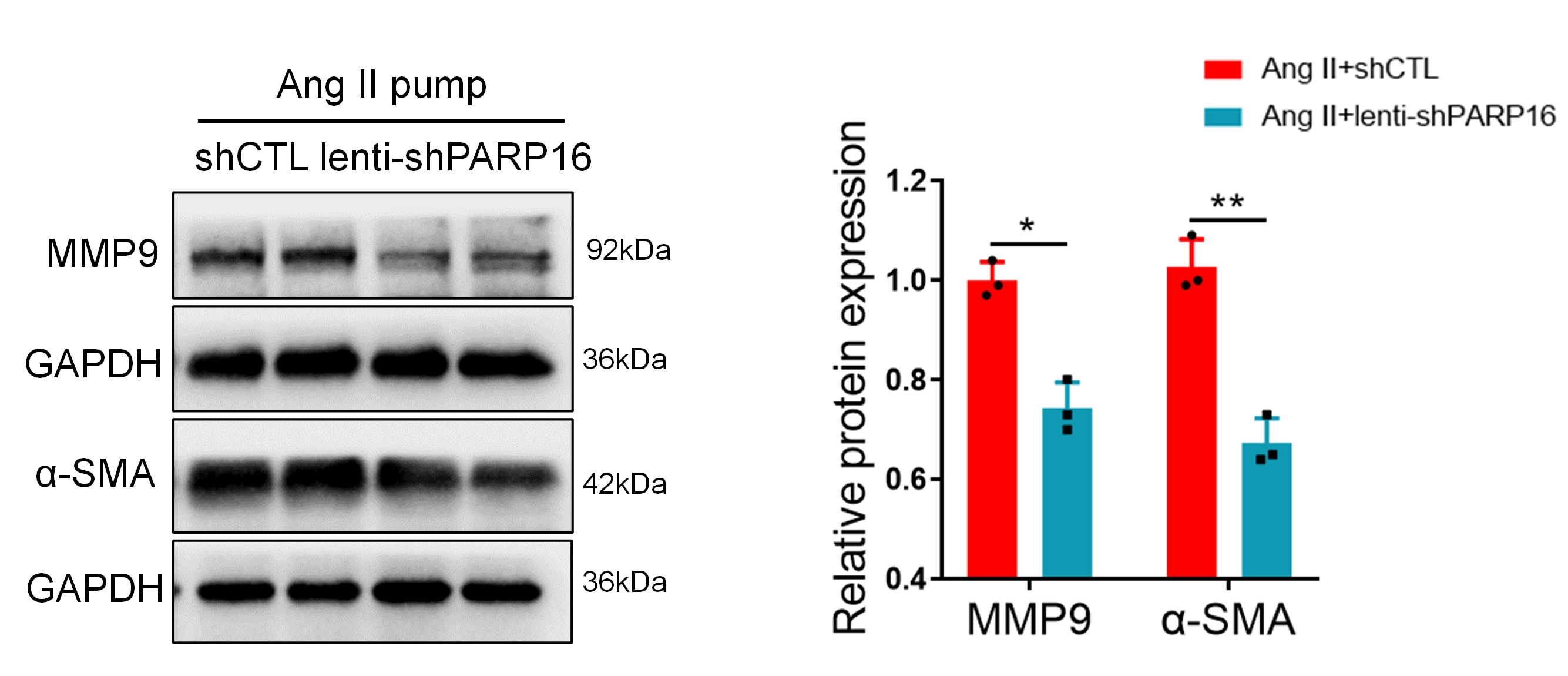
**

**Fig. S4 Knockdown of PARP16 prevents cardiac fibrosis in Ang II-infused cardiac hypertrophy mice model.** Mice received injection of either lentivirus PARP16 shRNA (shPARP16) or scramble (shCTL) every 5 days after the Ang II-infusion with mini-osmotic pumps for 28 days. The heart sections were harvested and assayed the protein expressions of fibrotic markers (MMP9 and α-SMA) by Western blot. Data are presented as mean ± SD, and analyzed using Student *t test*. n = 3. **p* < 0.05, ***p* < 0.01.


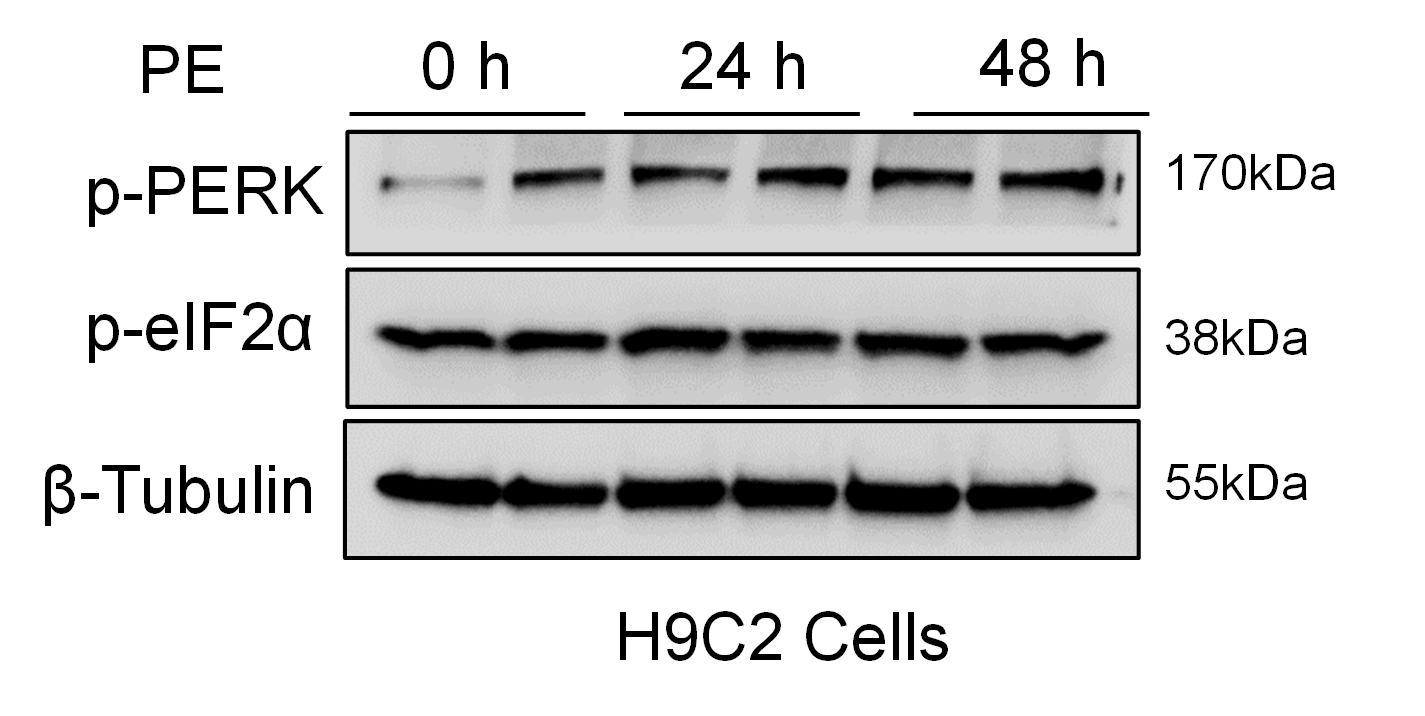


**Fig. S5 The PERK-eIF2α pathway was not disturbed upon PE treatment.** H9C2 cells were treated with PE (100 µM, 24 or 48 h) and then abstracted the protein to detect the p-PERK and p-eIF2α levels by western blot.


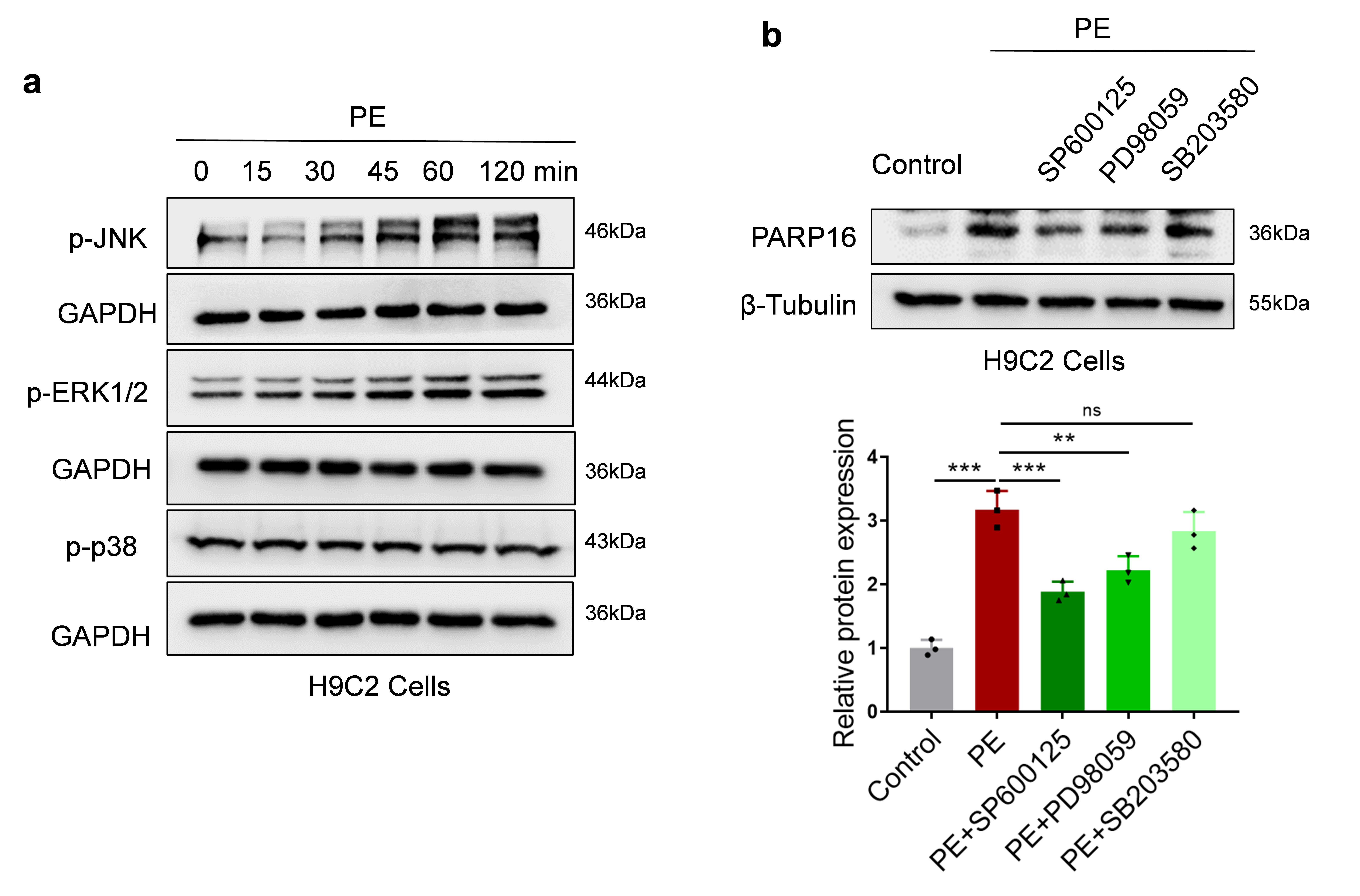


**Fig. S6 The expression of PARP16 is mediated by JNK/ERK MAPK pathway in PE-induced H9C2 cells.** (**a**) The phosphorylation of MAPK pathway members, including JNK, ERK1/2, except p38 MAPK were increased in PE-challenged H9C2 cells. (**b**) The expression of PARP16 was inhibited by JNK and ERK1/2 inhibitors but not p38 MAPK inhibitor in PE-challenged H9C2 cells. Data are presented as mean ± SD, and analyzed using one-way ANOVA followed by Tukey Post-hoc tests. n = 3. ** *p* < 0.01, ****p* < 0.001, ns = no significance.

**
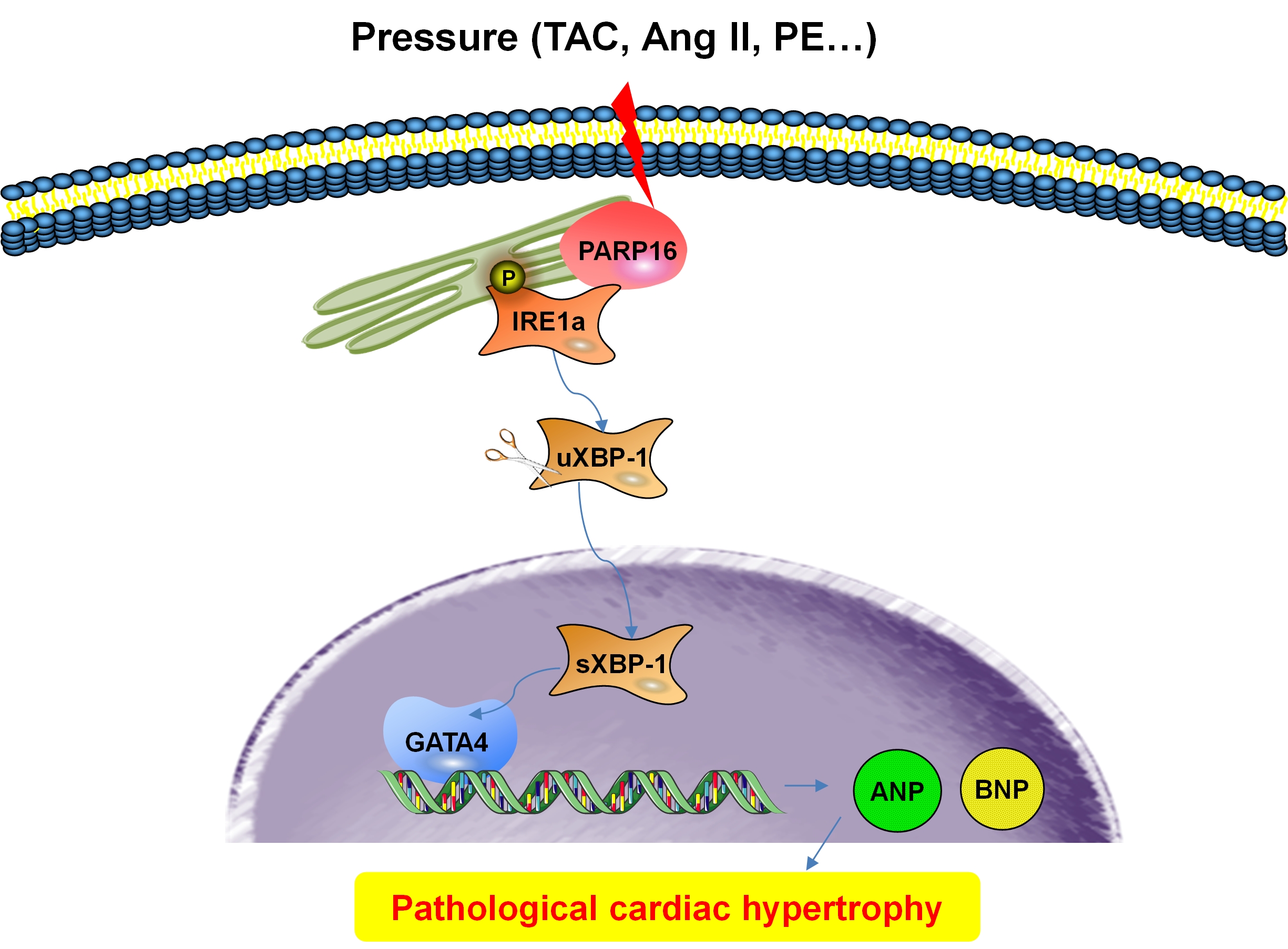
**

**Graphical Abstract. Schematic model of PARP16 regulates pathological cardiac hypertrophy.** PARP16 was significantly increased in response to TAC/Ang II-induced cardiac hypertrophy or PE-induced cardiomyocyte hypertrophic growth. The upregulated PARP16 interacted with IRE1α and ADP-ribosylated IRE1α to mediate the hypertrophic responses through activating the IRE1α-sXBP1-GATA4 pathway. PARP16 may be a new potential target for exploring effective therapeutic interventions for pathological cardiac hypertrophy and heart failure.

**SUPPLEMENTAL TABLE**

Table S1. The primer sequences used for qPCR analysis.

| **Gene** | **Forward (5′-3′)** | **Reverse (5′-3′)** |
| --- | --- | --- |
| Rat*-Nppa* | GGCACTTAGCTCCCTCTCTG | GGCAGATCTATCGGAGGGGT |
| Rat*-Nppb* | TTTCCTTAATCTGTCGCCGCT | TGCATCGTGGATTGTTCTGGA |
| Rat*-Myh7* | TTCGGGCGAGTCAAAGATGC | GCACTCGTCTTCCAGTTTGC |
| Rat*-Parp16* | CCGGCCAATGCCAGATTCTA | GCAGGCCGTTGTGAATGATG |
| Rat*-Gapdh* | TCAACGGCACAGTCAAGG | AGCATCAAAGGTGGAAGAAT |
| Rat*-Gata4* | TGAATGGTATCAACCGGCCC | TAGTCTGGCAGTTGGCACAG |
| Mouse-*Nppb* | CTGCTGGAGCTGATAAGAGA | TGCCCAAAGCAGCTTGAGAT |
| Mouse-*Fn* | AATCGTGCAGCCTCAATCCC | CAGGCTTGCTCTCGCAGTTA |
| Mouse*-Mmp9* | AAACCTCCAACCTCACGGAC | TTGGAATCGACCCACGTCTG |
| Mouse-*Ctgf* | ACCCAACTATGATGCGAGCC | TGCACACCCCGCAGAACTT |
| Mouse*-Gapdh* | CGACCACTTCGGCATTGTG | GGAGGCAGGGATGATGTTCT |

Table S2. Echocardiographic parameters in TAC-challenged mice treated with AAV9-shPARP16.

|  | Sham | TAC+AAV-GFP | TAC+AAV-shPARP16 |
| --- | --- | --- | --- |
| n | 9 | 11 | 11 |
| EF% | 61.18445±0.474387 | 39.55068±2.318502^***^ | 56.47845±1.343688^###^ |
| FS% | 32.01111±0.296923 | 19.09908±1.265525^***^ | 29.16329±0.886964^###^ |
| LV Mass,mg | 79.1565±4.245815 | 152.2788±6.77009^***^ | 108.1838±3.160253^###^ |
| LVESV,μl | 20.42435±1.58214 | 45.56373±2.997106^***^ | 29.317±1.507282^###^ |
| LVEDV,μl | 52.39602±3.688146 | 74.76913±2.772638^***^ | 67.4384±3.00916^ns^ |
| IVSd,mm | 0.688066±0.015933 | 1.066667±0.054198^***^ | 0.774074±0.019422^###^ |
| IVSs,mm | 1.095473±0.024954 | 1.375926±0.06816^**^ | 1.190741±0.033881^#^ |
| LVID;d,mm | 3.524074±0.10779 | 4.105556±0.064913^***^ | 3.927778±0.074665^ns^ |
| LVID;s,mm | 2.396914±0.077419 | 3.325926±0.090687^***^ | 2.781482±0.059511^###^ |
| LVPW;d,mm | 0.699383±0.016004 | 0.805556±0.052793^ns^ | 0.762963±0.021968^ns^ |
| LVPW;s,mm | 1.08642±0.02827 | 1.020371±0.04103^ns^ | 1.188889±0.0205^##^ |
| Heart Rate,bpm | 484.8164±15.48157 | 507.8726±16.89622 | 541.7875±18.23601 |

Data presented as the mean ± SEM and analyzed using one-way ANOVA followed by Tukey Post-hoc tests, ^**^*p*＜0.01, ^***^*p*＜0.001 compared to Sham, ^#^*p*＜0.05, ^##^*p*＜0.01, ^###^*p*＜0.001 compared to TAC, ns=no significance.

EF, ejection fraction %; FS, fractional shortening %; LVESV, left ventricle end-systolic volume; LVEDV, left ventricle end-diastole volume; IVSd, Interventricular Septum thickness at diastole; IVSs, Interventricular Septum thickness at systole; LVID; d, left ventricle internal dimension at diastole; LVID; s, left ventricle internal dimension at systole; LVPW; d, Left Ventricular Posterior Wall thickness at diastole; LVPW; s, Left Ventricular Posterior Wall thickness at systole.
